# Preventing Hip (Proximal Femoral) Fractures: An Evidence-Based Review for Clinicians

**DOI:** 10.3390/jcm15166148

**Published:** 2026-08-07

**Authors:** Toshiyuki Kawai, Yaichiro Okuzu, Yusuke Takaoka, Daichi Natsume, Shuichi Matsuda

**Affiliations:** Department of Orthopaedic Surgery, Graduate School of Medicine, Kyoto University, 54 Shogoin-kawahara-cho, Sakyo-ku, Kyoto 606-8507, Japan; yokuzu@kuhp.kyoto-u.ac.jp (Y.O.); ytakaoka@kuhp.kyoto-u.ac.jp (Y.T.); dnatsume@kuhp.kyoto-u.ac.jp (D.N.); smat522@kuhp.kyoto-u.ac.jp (S.M.)

**Keywords:** hip fracture, femoral neck fracture, prevention, FRAX, fracture liaison service, bisphosphonate, denosumab, romosozumab, teriparatide, vitamin D, calcium, hip protectors, falls, epidemiology, secular trends

## Abstract

Hip fractures are among the most devastating fragility fractures, associated with excess mortality, disability, loss of independence, and substantial healthcare costs. Although age-standardized incidence has declined in several high-income countries, absolute case numbers continue to rise because of population aging. This review summarizes contemporary PubMed-indexed evidence on the epidemiology, risk stratification, and prevention of proximal femoral fractures, with emphasis on hip-fracture outcomes rather than vertebral or composite endpoints alone. We discuss secular trends, FRAX-based case findings and screening, non-pharmacologic strategies, pharmacologic therapy, and health-system interventions relevant to both primary and secondary prevention. Among non-pharmacologic measures, long-term balance-challenging and resistance-based exercise has the most consistent evidence for reducing falls and likely contributes to fracture prevention, whereas multifactorial interventions, home hazard modification, calcium/vitamin D supplementation, and hip protectors are best targeted to selected high-risk populations and care settings. Among medications, bisphosphonates, denosumab, and romosozumab-based sequential strategies show the strongest evidence for reducing hip-fracture risk, while teriparatide and abaloparatide have important roles in very-high-risk patients despite less direct hip-fracture evidence. Menopausal hormone therapy reduces hip fractures in younger postmenopausal women but is limited by extra-skeletal risk, and selective estrogen receptor modulators are primarily vertebral-fracture agents. A major message of this review is that effective prevention depends not only on drug efficacy but also on implementation. Fracture liaison services, orthogeriatric co-management, prompt treatment after fragility fracture, and sustained adherence support are essential to close persistent care gaps. Preventing hip fractures therefore requires an integrated, risk-stratified approach that combines skeletal protection, falls prevention, and reliable health-system delivery.

## 1. Introduction

Hip fractures are among the most serious fragility fractures in older adults, and intracapsular femoral neck fractures represent an important subtype within this broader category. They are associated with excess mortality, persistent disability, loss of independence, institutionalization, and major healthcare costs. Recovery is often incomplete; compared with age-matched controls, hip fracture survivors have substantially worse long-term mobility, functional independence, and quality of life after injury [1,2]. Because these injuries reflect the combined effects of frailty, sarcopenia, osteoporosis, and multimorbidity, they are better understood as sentinel events in later life than as isolated orthopedic injuries [1,2].

Globally, the epidemiology of hip fracture has become increasingly complex. Age- and sex-standardized rates have declined in many high-income settings, but these gains have slowed or plateaued in some countries, and the absolute number of cases is still expected to rise because of population aging [2]. In a multinational analysis of more than 4.1 million hip fractures across 19 healthcare systems, incidence, post-fracture treatment, and one-year mortality varied markedly by region, while the total annual number of hip fractures was projected to nearly double between 2018 and 2050 [2]. This tension is especially relevant to Japan, one of the world’s most rapidly aging societies. Nationwide survey data have shown that although incidence may have declined in some age strata, particularly among people in their 70 s, the total number of hip fractures continued to increase through 2017 [3].

Mechanistically, hip fracture occurs when skeletal fragility intersects with exposure to falls. Most hip fractures are fall related, while osteoporosis, cortical thinning, deterioration of bone microarchitecture, sarcopenia, impaired balance, gait disorders, visual impairment, cognitive decline, polypharmacy, and environmental hazards all contribute to risk [4,5]. This has an important implication for prevention: hip fracture cannot be prevented by a single intervention or within a single specialty. Meaningful prevention therefore requires coordinated strategies that reduce the likelihood of falls while improving the ability of bone to resist impact [4,5,6].

Among non-pharmacologic approaches, structured exercise and targeted fall prevention are foundational. Contemporary international guidance and updated evidence syntheses support balance-challenging and multicomponent exercise as the most consistently effective strategy for reducing falls and fall-related morbidity in community-dwelling older adults [5,6]. However, fall prevention alone is unlikely to be sufficient in individuals with advanced skeletal fragility, emphasizing the need for integrated assessment and treatment.

Risk stratification has therefore become central to modern prevention models. FRAX-based case findings have improved the feasibility of identifying individuals at elevated hip-fracture risk, and the SCOOP trial demonstrated that community-based screening can reduce hip fractures in older women [7]. Current national guidance has moved toward integrated frameworks that combine clinical risk factors, bone mineral density when available, and intervention thresholds designed to support earlier and more proportionate treatment [8]. This approach is attractive because it allows preventive efforts to be directed toward patients most likely to benefit [7,8].

Effective pharmacologic therapies are also available, but their use remains inconsistent in real-world care. Contemporary network meta-analyses and guidelines support the ability of anti-osteoporosis medications to reduce hip and other major fragility fractures in appropriately selected patients [8,9]. Nevertheless, treatment gaps remain substantial after both first and subsequent fragility fractures. In the multinational study by Sing et al., post-hip-fracture treatment rates ranged from only 11.5% to 50.3% across healthcare systems, highlighting the persistent disconnect between evidence and implementation [2].

Secondary prevention models such as fracture liaison services (FLSs) were developed to address this problem by systematically identifying patients after fragility fracture, initiating osteoporosis evaluation and treatment, and supporting long-term persistence. Consensus recommendations place post-fracture coordination, timely anti-osteoporosis therapy, and fall-risk assessment at the center of secondary fracture prevention [10]. Recent systematic review evidence further indicates that FLS programs are associated with lower rates of secondary fragility fractures over time [11]. For clinicians, this means that prevention of hip fracture should be viewed not only as a question of drug efficacy or exercise prescription but also as a question of service delivery.

Despite this progress, hip-fracture prevention remains methodologically challenging. Many randomized trials and large observational studies use hip fracture as the primary endpoint rather than reporting intracapsular femoral neck fractures separately. Accordingly, throughout this review, hip fracture is used as the default evidence term for the composite proximal femur outcome reported in the literature, whereas femoral neck fracture is reserved for anatomically specific statements, femoral neck BMD findings, or studies that explicitly separate intracapsular fractures. We summarize recent epidemiologic trends [2], discuss approaches to risk assessment and screening [7], and critically review non-pharmacologic [6], pharmacologic [9], and health-system interventions [11] that may reduce the incidence of hip fractures, including femoral neck fractures.

## 2. Non-Pharmacologic Primary Prevention of Hip Fractures

Hip fractures in older adults are predominantly fall-related injuries occurring on a background of age-related skeletal fragility, sarcopenia, and impaired balance. In non-pharmacologic prevention trials, intracapsular femoral neck fractures are seldom reported separately from intertrochanteric or other proximal femur fractures. Accordingly, the most clinically relevant evidence usually has to be inferred from hip-fracture outcomes, injurious-fall outcomes, and validated intermediates such as gait, lower-extremity strength, balance, and femoral neck or total-hip bone mineral density (BMD). Accordingly, this section uses hip fracture as the principal clinical outcome term and refers to femoral neck measures when site-specific anatomic or densitometric information is available. Contemporary evidence therefore supports a hierarchical prevention model: structured exercise is the cornerstone; targeted multifactorial intervention is most useful in people at higher baseline risk; and environmental or institutional measures should be individualized according to setting, frailty, and adherence feasibility [4,12,13,14,15].

### 2.1. Exercise Interventions: The Strongest Evidence Base

Among non-pharmacologic strategies, exercise has the strongest and most consistent evidence base. Long-term exercise programs reduce overall falls and injurious falls, and they probably reduce fracture incidence as well, although most individual trials remain underpowered for hip fracture specifically [16]. In this context, consistent evidence syntheses support prioritizing exercise because they show reductions in falls, injurious falls, fall rate, and the likelihood of becoming a faller—upstream events most directly linked to proximal femoral fracture risk. The 2019 JAMA Internal Medicine meta-analysis showed significant reductions in falls and injurious falls [16], while the 2019 Cochrane review and the 2024 USPSTF evidence update confirmed that exercise lowers both fall rate and the likelihood of becoming a faller in community-dwelling older adults [6,17]. Current world falls guidelines and recent JAMA review articles accordingly place exercise at the center of primary fall-prevention care [14,15].

The efficacy of exercise depends on content and dose. Programs that challenge balance and progressively strengthen the lower limbs outperform non-specific activity advice or walking alone. Multicomponent regimens typically combine balance training, gait or functional task practice, sit-to-stand transfer training, and resistance exercise, delivered over months rather than weeks. Tai Chi has a credible evidence base for fall prevention, and recent systematic reviews suggest that structured home-based models such as the Otago Exercise Program can improve balance, mobility, and strength when progression and adherence are actively supported. Home-based exercise can also work after a sentinel fall if coaching is maintained, whereas dance-based interventions remain promising for engagement but currently lack evidence comparable to structured balance and resistance training. Serious exercise-related adverse events are uncommon when programs are individualized and appropriately supervised [17,18,19,20,21].

The clinical importance of exercise extends beyond short-term fall metrics. Karinkanta and colleagues reported durable reductions in injurious falls and fractures after a combined resistance, balance, and jumping intervention, and a more recent meta-analysis focusing on major osteoporotic fractures found a significant overall fracture reduction, particularly in longer and progressive programs. Exercise also improves biologic determinants of hip fracture risk: progressive loading and resistance training produce modest but meaningful gains in femoral neck and total hip BMD, and high-velocity resistance training may further enhance skeletal response in appropriately selected older adults. Observational data are consistent with this signal; in a 20-year cohort of older women, regular walking was associated with lower hip-fracture risk, although walking by itself should not be mistaken for a complete fall-prevention program [22,23,24,25,26].

An important limitation of this literature is that hip fracture remains a rare and usually secondary endpoint, so the case for exercise still rests more on convergent fall, injurious fall, and BMD data than on hip-powered fracture trials. This does not diminish the practical value of exercise, but it does mean that the magnitude of its direct hip-fracture effect remains uncertain. In addition, substantial heterogeneity in intervention content, supervision, progression, and adherence limits cross-trial comparability; accordingly, “exercise” should not be interpreted as a single uniform intervention in guideline-style terms [6,14,15,17,24].

### 2.2. Multifactorial Risk Assessment and Tailored Intervention

Multifactorial fall-prevention interventions remain important, but the evidence is more context-dependent than for exercise. These programs begin with an individualized assessment of gait and balance, medications, vision, orthostatic symptoms, cognition, footwear, continence, and environmental hazards, followed by tailored actions such as exercise prescription, home modification, medication adjustment, or referral. The classic Tinetti trial established the principle that coordinated risk-factor modification can reduce falls in high-risk community-dwelling older adults, and later systematic reviews confirmed that multifactorial programs can decrease fall rate when assessment is linked to actual implementation of interventions rather than advice alone [14,15,27,28,29].

However, recent evidence shows that multifactorial care is not a uniform solution. The 2024 USPSTF review and a contemporary *JAMA* review concluded that multifactorial interventions produce modest reductions in fall rate but less consistent effects on the proportion of fallers, injurious falls, or fracture endpoints than exercise alone. This heterogeneity probably reflects differences in uptake and intervention intensity. Programs that identify risks without ensuring supervised exercise, environmental correction, or meaningful deprescribing tend to underperform. The large pragmatic PreFIT trial underscores this limitation; population screening followed by targeted exercise or targeted multifactorial intervention did not reduce fractures compared with mailed advice alone [30]. In practice, multifactorial intervention is most defensible for older adults with prior falls, frailty, gait disorder, cognitive impairment, or other clear markers of elevated risk [14,15].

### 2.3. Environmental and Institutional Strategies

Environmental strategies are best viewed as targeted amplifiers of exercise-based prevention rather than stand-alone substitutes. The 2023 Cochrane review of environmental interventions and a 2023 systematic review of home hazard modification programs support the value of occupational therapy-led home assessment and hazard reduction in selected community-dwelling older adults, especially those with a history of falls or functional limitations. Yet contemporary trials also demonstrate the limits of universal home modification. Both the OTIS study and a JAMA Network Open trial of home hazard removal reduced hazards and improved some process or secondary outcomes, but their effects on falls were mixed, implying that environmental change alone may be insufficient when impairments in balance, strength, vision, cognition, or medication burden are not simultaneously addressed [31,32,33,34].

The balance of evidence differs in residential aged care and nursing homes, where baseline risk is higher, adherence can be externally supported, and staff practices shape outcomes. Umbrella reviews and Cochrane reviews indicate that multifactorial, staff-supported, or environmental interventions in care facilities can reduce falls more consistently than population-wide community programs, although heterogeneity remains substantial and not every exercise trial succeeds. A recent long-term care randomized trial of the Staying UpRight program reported no reduction in falls, probably in part because COVID-related interruptions markedly reduced delivered dose. Nurse-led prevention also matters: a 2024 systematic review found that nursing interventions, especially multifactorial and education-based approaches, reduced falls across community and health-care settings, emphasizing the importance of day-to-day implementation, monitoring, and follow-through [35,36,37,38,39].

### 2.4. Adjunctive Supportive Measures

Several adjunctive measures deserve brief comment. Medication review and deprescribing are mechanistically attractive, particularly for sedatives, psychotropics, and drugs that worsen orthostatic hypotension, but the current evidence suggests that medication review is more effective as one element of a broader program than as a stand-alone intervention. At the population level, broad community-based falls initiatives remain conceptually attractive, but a recent Cochrane review found that high-certainty evidence for reduced falls or fall-related injuries at scale is still lacking [36,40,41].

### 2.5. Clinical Synthesis

For clinicians seeking to prevent hip fractures, the practical message is clear. Because non-pharmacologic trials rarely separate femoral neck from other hip fractures, the recommendations below should be interpreted as hip-fracture prevention strategies with direct relevance to femoral neck fracture. The most evidence-based non-pharmacologic strategy is long-term, balance-challenging, resistance-based exercise offered as broadly as possible to at-risk older adults, combined with targeted home hazard modification, medication rationalization, and multidisciplinary or nursing follow-up for those with recurrent falls, frailty, cognitive impairment, or institutional residence [17,31,37,39]. Exercise has the strongest evidence for reducing falls and injurious falls and probably contributes to hip-fracture prevention through both fall reduction and modest improvements in femoral neck and total hip bone strength [16,23]. Multifactorial and environmental approaches are not unimportant; rather, their benefits depend heavily on selecting the right patients, delivering the recommended components, and maintaining adherence over time [37].

### 2.6. Summary

Taken together, the non-pharmacologic evidence supports a hip-fracture prevention strategy that prioritizes long-term balance-challenging, resistance-based exercise as the core intervention, because it most consistently reduces falls and injurious falls and likely contributes to fracture prevention through favorable effects on strength, balance, and hip/femoral-neck BMD. Multifactorial assessment, home hazard modification, and institution-specific strategies remain important, but their effectiveness is more context-dependent and is greatest when targeted to older adults with prior falls, frailty, gait impairment, cognitive vulnerability, or residential-care needs. Adjunctive measures such as medication review should therefore be viewed as supportive components within a broader, risk-stratified prevention program rather than as stand-alone substitutes for structured exercise.

## 3. Hip Protectors as a Non-Pharmacological Adjunct

Hip protectors are a non-pharmacological intervention, but they are discussed separately here because their effectiveness is highly setting-specific and depends heavily on adherence and care environment.

### 3.1. Rationale and Mechanism

Most hip fractures result from a lateral fall with direct impact over the greater trochanter; devices that shunt impact forces away from the proximal femur or absorb energy can, in principle, lower fracture risk. Modern hip protectors are typically “hard-shell” (energy-shunting) or “soft-shell” (energy-absorbing) pads integrated into garments or adhesive devices. Consensus standards emphasize laboratory verification of impact attenuation using biofidelic pelvis surrogates before clinical use, because performance varies widely across models and with pad misplacement [42].

### 3.2. Evidence from Randomized and Meta-Analytic Studies

The most comprehensive synthesis remains the Cochrane review (search to December 2012; 19 trials, ~17,000 participants). Pooled data from nursing/residential care show a probable reduction in hip fractures (RR 0.82, 95% CI 0.67–1.00; ≈11 fewer per 1000 persons/year), with no reduction in falls and a possible small increase in pelvic fractures (absolute ≈1 more per 1000). In community-dwelling adults, pooled trials show little to no effect (RR 1.15, 95% CI 0.84–1.58). Adverse effects were uncommon (skin irritation ≤5%). Adherence was low to moderate (≈24–80%) [43].

### 3.3. Findings of Individual Large Trials Are Mixed

An early Finnish randomized trial in frail elders reported a substantial risk reduction overall and when protectors were worn at the time of a fall [44]. Subsequent trials using more rigorous designs have been neutral, e.g., the multicenter HIP PRO trial in U.S. nursing homes (individuals served as their own controls with one padded hip): no difference in hip-fracture incidence between protected and unprotected hips despite protocol-measured adherence [45]. These discrepancies—together with meta-analytic results—support a cautiously favorable conclusion in institutional settings and a neutral conclusion in the community.

### 3.4. “Worn-At-the-Time-of-Fall” Evidence and Device Heterogeneity

Event-level analyses consistently show protection when a device is actually in place during a fall. In Norwegian nursing homes, hip-fracture odds were reduced to about one-third in protected versus unprotected falls; similar risk reductions (~60%) were observed for both soft and hard pads [46,47]. A more recent prospective study in long-term care similarly found an ≈3-fold reduction in fracture risk when protectors were worn at the moment of falling [48]. Conversely, laboratory testing and early clinical work highlight that performance varies among products and declines with pad misplacement—reinforcing the need for validated, well-designed protectors and correct fitting [42,49].

### 3.5. Adherence: The Principal Barrier

Across trials and implementation studies, acceptance and long-term adherence are the dominant determinants of effectiveness. Cochrane reported wide adherence ranges (≈24–80%) [43]. Systematic review evidence in long-term care identifies discomfort, aesthetics, toileting challenges, and workflow issues as key barriers; multicomponent strategies (no-cost provision, education, staff engagement, and user-friendly designs) can improve uptake but often do not sustain high wear time [50,51]. U.S. multicenter data show facility-level factors (e.g., staffing culture) correlate with adherence, suggesting targeting and operational support are required [52].

### 3.6. Safety

Skin irritation is uncommon (0–5%). The best available meta-analytic estimate suggests a possible small increase in pelvic fractures, but the absolute risk difference is very small (~1 per 1000) [43].

### 3.7. Cost-Effectiveness

Economic evaluations generally find hip protectors cost-effective in high-risk, institutional populations (e.g., long-term care) and potentially cost-saving to payers if adherence is adequate; cost-effectiveness is less certain in community settings with lower baseline risk and adherence [53,54].

### 3.8. Guideline Positions

Recommendations align with the evidence gradient by setting. Nursing and geriatric society guidance commonly considers hip protectors for frail older adults in long-term care as part of multifactorial fall-injury prevention while advising against routine use in the community. For example, the Registered Nurses’ Association of Ontario (RNAO) guideline recommends considering hip protectors for adults at risk of falls/hip fractures, and the RACGP/Osteoporosis Australia guideline recommends consideration in residential-care settings but not in community settings. A CADTH guideline synopsis reports similar mixed guidance for community dwellers (one suggestion to consider in select high-risk circumstances; one conditional recommendation for frail adults in appropriate environments; one recommending against in community) [55,56].

### 3.9. Implications for Practice (By Setting)

#### 3.9.1. Nursing-Home/Residential Care

- Offering well-tested hip protectors to ambulatory residents at high risk is reasonable. Program success depends on device selection (validated impact attenuation, good fit, ease for toileting), staff training, individualized consent, and adherence monitoring; integrating protectors within multifactorial injury-prevention bundles (vitamin D repletion where indicated, osteoporosis treatment, medication review, exercise/physiotherapy, and environmental modifications) is recommended [42,57].

#### 3.9.2. Community Dwellers

- Routine provision is not supported by randomized evidence; discussions may be individualized for exceptionally high-risk persons who accept daily wear (e.g., severe osteoporosis with recurrent lateral falls), but expectations should be cautious and adherence realities weighed. Priority should be given to proven measures (fall-risk assessment and mitigation, exercise/balance training, and guideline-concordant osteoporosis therapy) [43].

### 3.10. Summary

In nursing-home/residential settings, well-designed protectors probably reduce hip-fracture risk; real-world effectiveness hinges on achieving sustained wear. In community dwellers, randomized evidence is limited/neutral, so routine use is not recommended; consider only case-by-case in very high-risk individuals who are likely to adhere [43].

## 4. Vitamin D and Calcium

### 4.1. Scope and Definitions

Because randomized trials and meta-analyses rarely separate intracapsular (femoral-neck) from intertrochanteric fractures, this chapter treats “hip fracture” as the best available endpoint for proximal femoral (including femoral-neck) fracture risk. Where possible, setting (community vs. institutional) and regimen (daily vs. bolus dosing; with vs. without calcium) are specified.

### 4.2. Biological Rationale

Vitamin D promotes intestinal calcium absorption and maintains mineral homeostasis; calcium is required for skeletal mineralization. Deficiency of either increases secondary hyperparathyroidism, cortical bone loss (notably at the hip), and fall risk via myopathy—mechanisms that plausibly affect femoral-neck fracture risk. Yet clinical benefit depends on baseline status, dose, co-administration, and adherence [58].

### 4.3. Vitamin D Alone (Community-Dwelling Adults)

#### 4.3.1. Large Recent RCTs

The VITAL ancillary fracture trial (*n* = 25,871; 2000 IU/day cholecalciferol; median 5.3 years) showed no reduction in total, non-vertebral, or hip fractures (hip HR 1.01, 95% CI 0.70–1.47) and null effects across baseline 25(OH)D subgroups [59].

#### 4.3.2. Meta-Analytic Syntheses (Recent)

An umbrella review of RCT meta-analyses concluded that vitamin D alone does not lower hip or any fracture risk; discordant findings across reviews were explained by inclusion of small, biased, or institutional trials and by combining vitamin D with calcium [58].

The comprehensive 2018 Lancet Diabetes & Endocrinology meta-analysis similarly found no meaningful effects of vitamin D on fractures or falls and provided trial-sequential “futility” evidence for clinically important fracture reductions, including at the hip [60].

#### 4.3.3. Dosing Pattern Matters

Bolus regimens (e.g., 500,000 IU annually; 100,000 IU monthly) do not prevent fractures and may increase falls and/or fractures; a 2010 randomized trial in older women reported higher falls and fractures with 500,000 IU once-yearly cholecalciferol, and large monthly trials showed null effects on fractures. A 2023 analysis of monthly dosing reassured that fracture risk is not increased overall but still failed to show benefit [61,62,63].

#### 4.3.4. New Evidence Signal (Women)

A 2024 meta-analysis of “healthy elderly” RCTs (*n* ≈ 71,900) found no effect of vitamin D on total fractures but reported a higher hip-fracture risk among women (RR 1.34, 95% CI 1.06–1.70). This reinforces caution about prescribing vitamin D without calcium to replete adults solely for fracture prevention [64].

#### 4.3.5. Bottom Line for Vitamin D Alone

Across modern, well-powered trials in vitamin D-replete, community-dwelling adults, vitamin D monotherapy does not prevent hip or femoral-neck fractures.

### 4.4. Calcium Alone

Systematic reviews indicate small, inconsistent effects of calcium supplements on total fractures and no convincing reduction in hip fractures; BMD gains are modest (~1% at the hip) and plateau early, limiting expected fracture benefit [65,66].

### 4.5. Combined Vitamin D + Calcium (Ca/D)

#### 4.5.1. Institutionalized (Nursing-Home/Residential) Populations

Classic, high-adherence trials in very old, institutionalized women with low calcium/vitamin D intakes showed clinically important hip-fracture reductions with Ca/D (800 IU cholecalciferol + ~1200 mg calcium daily). In Decalyos I ([67]; mean age 84), hip fractures fell by 43% over 18 months; Decalyos II confirmed benefit signals and reduced femoral-neck bone loss. These trials also corrected secondary hyperparathyroidism [67,68].

#### 4.5.2. Community-Dwelling Adults

Evidence is mixed and sensitive to adherence and dose. The Women’s Health Initiative (WHI) CaD trial (1000 mg calcium carbonate + 400 IU vitamin D3 daily) did not significantly lower hip-fracture risk overall, though adherence-restricted analyses suggested a modest benefit; nephrolithiasis increased. Parallel USPSTF evidence syntheses similarly concluded no net benefit in community-dwelling adults without deficiency [69].

#### 4.5.3. Recent Quantitative Syntheses

A 2019 meta-analysis (JAMA Network Open) that examined both observational gradients in 25(OH)D and trial effects reported no fracture reduction from vitamin D alone and mixed/weak effects for Ca/D overall. More recent reviews highlight that any fracture reduction with Ca/D is most evident when institutional and community trials are combined—driven largely by the institutional trials with low baseline intake and high adherence [70].

Some newer meta-analyses suggest Ca/D can reduce hip fracture risk when daily vitamin D is ~800–1000 IU and calcium ≥1200 mg with good adherence, but these effects attenuate or vanish in community-only datasets [71].

#### 4.5.4. Practical Inference

Ca/D shows its clearest hip-fracture benefit in frail, institutionalized elders with low baseline intakes. For community-dwelling, vitamin D-replete older adults, Ca/D has at best modest effects that are contingent on adherence and sufficient dosing and are offset by kidney-stone risk in some settings [67,69].

A central controversy in this field is that apparently discordant trial results often reflect differences in baseline deficiency, calcium intake, residence, adherence, and dosing schedule. They do not necessarily indicate a simple yes-or-no effect of supplementation. This makes broad recommendations difficult: the clearest benefit signal is confined to frail institutionalized populations, whereas evidence in vitamin D-replete community dwellers is weak and sometimes offset by harms. An unresolved question is whether a clinically identifiable subgroup of community-dwelling older adults can derive meaningful hip-fracture benefit from targeted Ca/D supplementation without an unfavorable balance of nephrolithiasis or overtreatment [58,67,69,70,72].

### 4.6. Guideline Positions (2024–2025)

- USPSTF (Draft, 17 December 2024): Recommends against vitamin D supplementation—with or without calcium—for the primary prevention of fractures (and against vitamin D to prevent falls) in community-dwelling postmenopausal women and men ≥60 y (Grade D) [73].
- NOGG (UK 2024): Ensure adequate dietary calcium and vitamin D in all, and supplement as needed; treat deficiency and co-administer Ca/D as adjuncts when initiating anti-osteoporosis drugs or when intake is low—i.e., as part of comprehensive management, not stand-alone fracture prophylaxis [8,74].

### 4.7. Safety, Dosing, and Implementation Considerations

- Avoid high-dose bolus strategies. Annual 500,000 IU cholecalciferol increased falls and fractures in older women; large monthly dose trials showed no benefit for fractures. If supplementing, prefer daily dosing [61,62,63].
- Kidney stones. Ca/D increased nephrolithiasis in WHI; USPSTF reviews flag this harm signal when weighing net benefit [8,69,75].
- Target those likely to benefit. Correct documented vitamin D deficiency and inadequate calcium intake, particularly in institutional/residential settings and prior to parenteral antiresorptives; ensure adherence [8,75].

### 4.8. Synthesis for Femoral-Neck (Hip) Fracture Prevention

1. Vitamin D alone does not prevent hip (femoral-neck) fractures in community-dwelling, vitamin D-replete adults; signals of harm emerge with high bolus dosing and possibly among healthy elderly women [59,60,64].
2. Calcium alone yields small, inconsistent fracture effects and negligible hip-fracture benefit, despite minor BMD gains [65,66].
  - Calcium + vitamin D reduces hip-fracture risk most convincingly in institutionalized elders with low baseline intakes; effects in community dwellers are small to neutral and depend on dose and adherence—and must be balanced against kidney-stone risk. Current preventive guidelines do not endorse routine Ca/D solely to prevent fractures in community-dwelling older adults without deficiency [67,72,73].

### 4.9. Summary

In summary, the evidence does not support vitamin D alone as a stand-alone strategy for preventing hip fractures in community-dwelling, vitamin D-replete older adults, and calcium alone has at most small skeletal effects with no convincing hip-fracture benefit. The clearest signal of benefit is for combined calcium plus vitamin D in frail, institutionalized older adults with low baseline intake or deficiency, whereas effects in community-dwelling populations are modest, adherence-dependent, and offset in some settings by nephrolithiasis risk. Clinically, calcium and vitamin D should therefore be used selectively—to correct deficiency, low intake, or treatment-related needs—rather than as universal stand-alone prophylaxis for hip-fracture prevention, and high-dose bolus vitamin D regimens should be avoided.

## 5. Pharmacologic Prevention (Focus on Hip-Fracture Endpoints)

Table 1 summarizes the hip-centered efficacy of antiresorptive and osteoanabolic therapies.

### 5.1. Bisphosphonates and Hip-Fracture Prevention

#### 5.1.1. Introduction and Mechanistic Rationale

Bisphosphonates are antiresorptive agents that bind avidly to hydroxyapatite, concentrate at sites of active remodeling, and suppress osteoclast-mediated bone resorption. By lowering bone turnover, they increase bone mineral density (BMD), improve bone microarchitecture, and reduce the likelihood that age-related trabecular and cortical deterioration will culminate in fragility fracture. Because most treatment trials report hip fracture as the composite proximal femur endpoint rather than isolated femoral neck fracture, the central clinical question is not whether bisphosphonates preserve BMD but whether they reduce hip fractures and, by extension, the broader hip-fracture burden in patients at meaningful baseline risk [9,80,108,109,110].

For hip-fracture prevention, the strongest randomized evidence is for alendronate, risedronate, and zoledronic acid. In contrast, ibandronate has consistently shown vertebral efficacy but has not established a similarly robust hip-fracture benefit [111,112]. Contemporary guidelines therefore continue to place these three agents at the center of first-line antiresorptive therapy for most patients with osteoporosis who do not require or cannot access an anabolic-first strategy [108,109,110,113].

#### 5.1.2. Evidence from Landmark Randomized Trials

The pivotal alendronate data came from the Fracture Intervention Trial (FIT). In women with prevalent vertebral fractures, alendronate reduced hip fractures by approximately one-half over 3 years, alongside major reductions in vertebral and other clinical fractures. In the parallel FIT cohort without prior vertebral fracture, the overall hip-fracture reduction was not statistically significant, but women who met densitometric criteria for osteoporosis derived clearer benefit. This distinction remains clinically important: bisphosphonates work best when absolute fracture risk is high enough for a measurable benefit to emerge over the time horizon of a trial [76,77,114].

Risedronate produced a similar pattern in the Hip Intervention Program. Among women aged 70–79 years with osteoporosis or very low femoral-neck BMD, risedronate significantly reduced hip fractures, whereas efficacy was less clear in those aged 80 years or older who had been enrolled primarily on the basis of non-skeletal risk factors such as falls risk. The lesson from HIP is not that advanced age negates treatment effect but that age alone is a poor surrogate for skeletal fragility if BMD or prior fragility fracture is not also taken into account [79,115].

Zoledronic acid provides the most compelling intravenous data. In the HORIZON-Pivotal Fracture Trial, once-yearly zoledronic acid reduced hip fractures by 41% over 3 years while also substantially reducing vertebral and non-vertebral fractures. In the HORIZON-Recurrent Fracture Trial, zoledronic acid given after surgical repair of a recent hip fracture reduced new clinical fractures by about one-third and was also associated with lower mortality. These findings are particularly relevant to hip-fracture prevention because they show benefit both before the first hip fracture and after a sentinel hip-fracture event [80,82].

Additional trials expanded the population in whom zoledronate may be useful. In men with osteoporosis, zoledronic acid clearly reduced vertebral fractures and improved BMD, although hip-fracture events were too few for definitive male-specific hip-fracture estimates. In older women with osteopenia, infusions every 18 months reduced overall fragility fractures, showing that selected patients with osteopenia but substantial age-related risk can still benefit from potent bisphosphonate therapy. Importantly, however, that osteopenia trial was not designed to prove a stand-alone reduction in hip fractures, so it should be interpreted as evidence for broader fracture prevention rather than definitive femoral-neck-specific efficacy in low-risk populations [81,116].

#### 5.1.3. Contemporary Evidence Synthesis

Recent evidence syntheses have strengthened, not weakened, the case for bisphosphonates in hip-fracture prevention. A 2022 meta-analysis focused specifically on hip fractures found an overall relative risk reduction of roughly one-third, with the most consistent signals for alendronate and zoledronic acid and a somewhat smaller but still clinically meaningful effect for risedronate. Large network meta-analyses likewise continue to show that bisphosphonates reduce hip, non-vertebral, and vertebral fractures versus placebo and remain competitive with other antiresorptive strategies, even if anabolic agents may rank higher for some fracture outcomes in very-high-risk patients [9,78,111,112].

The most practice-shaping recent synthesis may be the ACP living systematic review and network meta-analysis, which concluded that bisphosphonates and denosumab reduce hip, clinical, and vertebral fractures in primary osteoporosis and low bone mass. In parallel, the ACP living guideline reaffirmed bisphosphonates as the preferred initial pharmacologic therapy for postmenopausal women with primary osteoporosis and suggested them as initial therapy in men. The 2022 BHOF/NOF clinician’s guide, the 2020 AACE/ACE guideline, and the 2024 ASBMR/BHOF task-force statement are directionally aligned: for most patients at high hip-fracture risk, bisphosphonates remain the foundational antiresorptive option because they combine efficacy, familiarity, long-term data, and reasonable cost [9,108,109,110,113].

The updated Cochrane reviews are also useful because they reinforce an important nuance for clinicians writing treatment algorithms: the apparent magnitude of benefit depends heavily on whether the treated population resembles a primary-prevention cohort or a secondary-prevention cohort. In other words, when baseline risk is low, the number of hip fractures accrued during follow-up may simply be too small for an individual trial to detect a difference, even if the drug is biologically effective [114,115].

#### 5.1.4. Which Patients Derive the Clearest Hip-Fracture Benefit?

The patients most likely to gain a clinically meaningful reduction in hip-fracture risk are those with established osteoporosis, prior fragility fracture, advanced age plus low BMD [76,78,79], or a high FRAX probability [108,109,110]. Across trials and meta-analyses, prior vertebral fracture and very low femoral-neck BMD repeatedly identify cohorts in whom absolute and relative benefits are both most evident [76,78,79]. This is why current guidelines prioritize bisphosphonates for patients with T-scores ≤ −2.5, prior hip or vertebral fracture, or high fracture probability, rather than for all women with mild osteopenia [108,109,110].

That said, recent zoledronate studies have broadened thinking about whom to treat. Follow-up of the Reid osteopenia trial to 10 years suggested that fracture protection can persist well beyond the active treatment phase. Even more recently, Bolland and colleagues showed that infrequent zoledronate dosing in women aged 50–60 years can reduce morphometric vertebral fracture over long follow-up, highlighting the durability of bisphosphonate skeletal effects. These newer studies do not replace the classic hip-fracture RCTs, but they do support a more individualized view in which treatment intervals and duration may be tailored to baseline risk, age, and the pharmacologic persistence of the chosen agent [117,118].

Special populations deserve brief comment. In men, bisphosphonates are widely extrapolated to reduce hip-fracture risk because their effects on BMD and vertebral fracture align with those seen in women, although male trials are underpowered for hip endpoints. In mild-to-moderate chronic kidney disease, recent patient-level meta-analytic data suggest that efficacy is largely preserved and safety is not dramatically different from the general osteoporosis population, but evidence remains sparse in advanced CKD, where treatment decisions should be individualized and renal-specific contraindications respected [108,109,110,116,119].

#### 5.1.5. Secondary Prevention After a Hip Fracture

From the perspective of preventing a second hip fracture, the post-hip-fracture period is one of the most important treatment windows in osteoporosis care. HORIZON-Recurrent Fracture showed that zoledronic acid initiated after repair of a hip fracture reduces subsequent clinical fractures and improves survival, strongly supporting prompt evaluation and treatment in fracture liaison pathways. Contemporary secondary-prevention consensus recommendations similarly emphasize that a hip or vertebral fragility fracture should trigger not only rehabilitation and fall-prevention measures but also osteoporosis medication unless contraindicated [10,82].

A common reason for delayed treatment after hip fracture is concern about fracture healing. That concern has been repeatedly overstated. Randomized and observational data indicate that early bisphosphonate initiation—particularly with zoledronic acid after fixation of intertrochanteric or other fragility hip fractures—does not meaningfully impair healing or functional recovery in most patients. Thus, deferring treatment for many months after surgery is usually unnecessary and risks losing a period of exceptionally high imminent fracture risk [82].

#### 5.1.6. Duration of Therapy, Persistence of Effect, and Drug Holidays

How long to continue therapy is highly relevant in a review focused on hip-fracture prevention, because the same properties that make bisphosphonates attractive—skeletal binding and persistence—also allow periodic reassessment and, in selected lower-risk patients, a temporary drug holiday. FLEX showed that women who discontinued alendronate after 5 years lost some BMD and had more clinical vertebral fractures than those who continued, although differences in non-vertebral fracture were less pronounced. Extension studies of HORIZON-PFT similarly showed that continuing zoledronic acid beyond 3 years preserves BMD and may benefit those at higher ongoing fracture risk, whereas many lower-risk patients can stop after 3 to 6 years with residual benefit persisting for some time [120,121,122,123].

Recent mechanistic and clinical studies help explain why zoledronate in particular lends itself to intermittent dosing. A single infusion has measurable antiresorptive effects for years, and long-term follow-up from osteopenia trials indicates that antifracture benefit may outlast the treatment phase. These data have renewed interest in infrequent dosing regimens, but they should not be oversimplified into a one-size-fits-all approach. Patients at very high hip-fracture risk—especially those with recent hip or vertebral fracture, ongoing glucocorticoid exposure, or persistently very low femoral-neck BMD—are often better served by continued treatment or a planned transition to another active agent rather than a holiday [117,118,120,124].

In practice, most guidelines recommend reassessing fracture risk after about 5 years of oral bisphosphonate therapy or 3 years of intravenous zoledronic acid. A holiday is most reasonable for patients whose fracture risk has fallen to low or moderate levels; it is far less appropriate for those with a recent fragility fracture or persistently high hip-fracture probability. The clinician’s task is not to follow a calendar rigidly but to balance residual benefit, current risk, and the rare long-term adverse events associated with cumulative exposure [108,109,110,113,120].

#### 5.1.7. Safety, Adherence, and Real-World Effectiveness

For indicated patients, the benefit–risk balance remains strongly favorable. The principal long-term safety concerns are atypical femoral fracture and osteonecrosis of the jaw, both of which are rare but increase with treatment duration. Importantly, a large benefit–risk analysis estimated that many more typical osteoporotic fractures are prevented than atypical femoral fractures caused, particularly in older women at high baseline risk. A recent Danish case-cohort study using blinded radiographic review reinforced that atypical femoral fracture is a real duration-related signal but still an uncommon one in absolute terms. Clinically, the correct response is not to avoid bisphosphonates indiscriminately; it is to reassess need for continued therapy, investigate new thigh or groin pain promptly, and consider full-length femoral imaging when symptoms or prolonged exposure raise concern [9,120,125,126].

Effectiveness in routine care is also shaped by adherence. Oral bisphosphonates are limited by dosing inconvenience and gastrointestinal intolerance in some patients, while intravenous zoledronic acid largely solves adherence but can cause transient acute-phase reactions and requires attention to renal function and calcium/vitamin D status. A recent target-trial emulation of HORIZON-PFT found that the protective effect of zoledronic acid against hip fracture in real-world claims data tracked closely with the randomized trial over the early follow-up period, but diminished adherence in practice restricted the ability to sustain long-term effectiveness estimates. This is a crucial implementation message for clinicians: prescribing a bisphosphonate is not enough; ensuring administration, persistence, and follow-up is part of fracture prevention itself [80,109,110,127].

#### 5.1.8. Summary

Bisphosphonates remain central to hip-fracture prevention. The totality of evidence—from landmark RCTs [76,79,80,82], contemporary meta-analyses, updated Cochrane reviews, living guidelines [9,78,108], long-term extension studies, and recent real-world analyses—supports the conclusion that alendronate, risedronate, and zoledronic acid reduce hip-fracture risk most clearly in patients with osteoporosis or otherwise high absolute fracture risk. Their benefits are most convincing in secondary prevention and in patients with low femoral-neck BMD, but newer data also show durable skeletal effects that can inform individualized interval and duration strategies [117,118].

For clinicians, the practical implication is straightforward: identify high-risk patients early, initiate bisphosphonate therapy promptly when indicated [108,109,110,113], continue long enough to capture hip-fracture benefit, and reassess duration with attention to both ongoing fracture risk and rare long-term adverse effects [120,125]. When used in this risk-stratified fashion, bisphosphonates remain one of the most evidence-based and clinically impactful interventions available for preventing hip fracture [108,109,110,113,128].

### 5.2. Denosumab

#### 5.2.1. Mechanism, Dosing, and Clinical Question

Denosumab is a fully human monoclonal antibody against receptor activator of nuclear factor kappa-B ligand (RANKL) that suppresses osteoclast differentiation, activation, and survival. For osteoporosis, it is administered as 60 mg subcutaneously every 6 months. Because randomized trials rarely report intracapsular femoral neck fractures separately, the practical clinical question is whether denosumab reduces hip fracture as the composite proximal femur outcome and improves structural determinants of femoral neck strength. In this section, hip fracture is therefore the primary efficacy term, whereas femoral neck is used for site-specific BMD and structural analyses. In practice, inference about femoral neck fracture prevention is drawn from hip-fracture outcomes in randomized trials together with site-specific bone mineral density (BMD) and structural analyses at the femoral neck and total hip [83,84,86,129].

#### 5.2.2. Evidence from Randomized Controlled Trials

The pivotal FREEDOM trial is the foundation of the evidence base. In 7808 postmenopausal women with osteoporosis followed for 36 months, denosumab significantly reduced new vertebral, non-vertebral, and hip fractures versus placebo; hip fracture risk was reduced by about 40%, with absolute event rates of roughly 0.7% versus 1.2% [83]. This remains the highest-quality randomized evidence that denosumab protects the proximal femur.

A prespecified/hierarchical post hoc analysis of FREEDOM showed larger absolute hip-fracture benefits in women aged 75 years or older and in those with baseline femoral neck T-scores of −2.5 or less, supporting particular relevance in patients at the highest risk of femoral neck failure [87].

The Japanese DIRECT trial extended the evidence base to an Asian population and showed significant vertebral-fracture reduction over 24 months in women and men with osteoporosis, with hip and other non-vertebral fracture outcomes directionally consistent with FREEDOM but limited by small event counts [130]. In other supportive randomized settings, denosumab reduced vertebral fractures in men receiving androgen-deprivation therapy for non-metastatic prostate cancer, although hip events were too infrequent for firm conclusions [131]. Likewise, in glucocorticoid-induced osteoporosis, denosumab produced larger gains in total-hip and femoral-neck BMD than risedronate, but those trials were not powered for hip-fracture endpoints [132].

#### 5.2.3. Contemporary Evidence Synthesis

Recent evidence syntheses reinforce denosumab as an effective antifracture therapy while also clarifying where it sits relative to anabolic agents. The American College of Physicians living systematic review and network meta-analysis published in 2023, and its 2024 update alert, continued to support denosumab as an effective option for fracture prevention in primary osteoporosis without materially changing the overall conclusions during surveillance [9]. A large 2023 BMJ network meta-analysis similarly supported denosumab’s efficacy versus placebo, but anabolic therapies generally ranked higher for broader clinical-fracture outcomes in very-high-risk patients [112]. Clinically, this means that denosumab is clearly effective for hip-fracture reduction, yet anabolic-first strategies may be preferable when the goal is the fastest or largest reduction in imminent fracture risk [9,112,113].

#### 5.2.4. Comparative Effectiveness in Routine Practice

Direct fracture-endpoint randomized trials comparing denosumab with oral bisphosphonates are lacking, so comparative claims rely mainly on observational emulations. In a large US Medicare active-comparator study, denosumab initiation was associated with a 36% lower hip-fracture risk than alendronate in postmenopausal women with osteoporosis [133]. A Taiwanese population-based cohort study also suggested lower hip-fracture rates among persistent denosumab users relative to alendronate users [134]. However, head-to-head observational evidence is not entirely one-directional: a large South Korean claims-based study found denosumab and bisphosphonates had broadly comparable risks of total fracture and major osteoporotic fracture after propensity matching [135]. Taken together, the real-world literature is encouraging for denosumab, especially against alendronate, but remains vulnerable to residual confounding and treatment channeling. It therefore complements rather than supersedes the placebo-controlled randomized evidence [133,134,135].

A major limitation of the comparative denosumab literature is that its apparent advantage over oral bisphosphonates is derived largely from observational emulations rather than head-to-head fracture-endpoint randomized trials [133,134]. These studies are clinically informative, but they remain vulnerable to residual confounding, treatment channeling, and differential persistence, particularly because denosumab is often selected for patients who differ systematically from alendronate users. For femoral neck fracture specifically, the evidence also remains partly indirect, because the inference depends on composite hip-fracture outcomes [133,134] plus densitometric and structural data [84,86,129] rather than trials designed around isolated intracapsular fractures.

#### 5.2.5. Femoral Neck-Specific Considerations: BMD, Strength, and Treatment Targets

Because most studies report hip fracture as a composite endpoint, inference about isolated femoral neck fracture depends on densitometric and structural data. Denosumab produces progressive gains in total-hip and femoral-neck BMD during continued treatment, and these gains continue beyond the first 3 years in extension data [83,85]. In the FREEDOM program, higher achieved total-hip T-scores during denosumab therapy were associated with lower subsequent non-vertebral fracture risk, with a clinically meaningful flattening of risk as the total-hip T-score approached roughly −1.5 [84]. Finite-element analysis of FREEDOM participants showed that denosumab improves estimated hip and vertebral strength, supporting biomechanical plausibility for hip-fracture protection [86]. High-resolution imaging studies additionally showed reduced cortical porosity in the proximal femoral shaft with denosumab, changes that likely contribute to improved femoral strength [129]. In a recent randomized trial in frail older adults living in long-term care, denosumab significantly improved total-hip BMD over 24 months, suggesting that hip benefits extend to populations often excluded from pivotal trials [136]. Collectively, these data make it reasonable to infer that denosumab’s favorable effects on “hip fracture” are highly relevant to femoral neck fracture prevention [84,86,129,136].

A treat-to-target framework may further refine the use of denosumab. Recent task-force and modeling analyses emphasize that achieving higher hip BMD targets is associated with lower fracture risk, although the probability of reaching a given target depends strongly on baseline T-score and treatment duration [113,137]. For clinicians focused on proximal femoral fracture prevention, serial total-hip or femoral-neck BMD can therefore be used not only to confirm response but also to guide sequencing decisions when risk remains high [84,113,137].

#### 5.2.6. Duration and Long-Term Efficacy While on Treatment

Long-term denosumab therapy remains one of its major strengths. In the FREEDOM extension, continuous treatment for up to 10 years produced progressive BMD gains at the spine and hip with persistently low fracture rates and a low incidence of serious skeletal adverse events [85]. A post hoc virtual-placebo/FRAX-based analysis also suggested that long-term denosumab likely preserves substantial antifracture efficacy over time rather than merely maintaining BMD numerically [138]. For patients at sustained high hip-fracture risk, these data support continued treatment as long as injection timing is reliable and a discontinuation strategy is in place [85,138].

#### 5.2.7. Discontinuation, Delayed Dosing, and Sequential Therapy

The major limitation of denosumab in clinical practice is not lack of efficacy on treatment but the reversibility of effect after stopping. Discontinuation leads to rapid bone-turnover rebound, brisk loss of hip and spine BMD, and a markedly increased risk of multiple vertebral fractures [88,139,140,141]. Although the rebound signal is strongest at the spine, rapid loss at the total hip and femoral neck is also highly relevant when the goal is proximal femur protection in frail older adults.

Recent data further sharpen this implementation message. A large population-based study from Korea found that even a 1-to-3-month delay beyond the recommended dosing window was associated with increased fracture risk, and longer delays were worse [89]. Accordingly, current guidelines emphasize that denosumab injections should not be missed or deferred without an alternative antiresorptive plan [8].

Sequential therapy after denosumab remains necessary but imperfect. Transition to alendronate can maintain much of the BMD gained during shorter denosumab exposure in many patients [142]. However, more recent randomized and cohort data suggest that zoledronic acid does not fully prevent post-denosumab bone loss in all patients, especially after longer denosumab duration or in those with persistently elevated bone turnover; even repeated zoledronate infusions 6 months apart may be insufficient in some cases [143,144]. The practical message is that denosumab should be prescribed only when a reliable long-term administration and exit strategy is feasible [8,88,142,143,144].

#### 5.2.8. Renal Impairment

Denosumab is often attractive when renal impairment limits bisphosphonate use. Post hoc analyses of FREEDOM found broadly similar fracture and BMD benefits across mild to moderate chronic kidney disease strata [145], and extension data suggested maintained efficacy and acceptable safety in stage 2–3 chronic kidney disease [146]. The key caveat is hypocalcemia risk, which rises as renal function worsens; calcium and vitamin D sufficiency and biochemical monitoring are therefore essential, particularly in advanced chronic kidney disease [145,146].

#### 5.2.9. Guideline Positioning

Contemporary guidelines place denosumab among the major antiresorptive options for adults at high fracture risk while strongly warning against unscheduled interruption. The 2024 UK guideline and recent goal-directed task-force statements recommend choosing denosumab when an effective antiresorptive is needed and reliable 6-month dosing can be assured but favor an anabolic-first approach for some very-high-risk patients who need more rapid or larger risk reduction [8,113]. These same sources also emphasize treatment targets at the hip and planned sequencing—especially when denosumab may eventually need to be stopped [8,113,137].

#### 5.2.10. Bottom Line for Hip-Fracture Prevention and Femoral-Neck Relevance

1. Denosumab has strong randomized evidence for hip-fracture reduction versus placebo, and subgroup analyses suggest particularly meaningful absolute benefits in very old adults and in patients with low baseline femoral-neck BMD [83,87].
2. For “femoral neck” specifically, the evidence is indirect but persuasive: hip-fracture reduction is accompanied by consistent gains in femoral-neck/total-hip BMD [136], improved estimated hip strength [86], reduced cortical porosity [129], and favorable treat-to-target relationships with achieved hip T-scores [84,113,137].
3. In comparative use, denosumab is at least a strong alternative to oral bisphosphonates and may outperform alendronate for hip fracture in some real-world datasets, although observational results are not completely uniform [133,134,135].
4. The principal clinical hazard is delayed dosing [89] or unplanned discontinuation [88,139,140]. Denosumab is therefore most suitable when the care team can guarantee on-time injections [89] and a sequential antiresorptive strategy at the time of cessation [8,142,143,144].

Overall, for older adults at high risk of hip fracture—especially those who cannot adhere to oral therapy or have contraindications to bisphosphonates—denosumab is a robust option for hip-fracture prevention. Its value for femoral neck fracture prevention is best viewed as a combination of direct hip-fracture evidence and convergent femoral-neck/hip structural evidence. The treatment works very well while it is given on time; the central implementation challenge is ensuring that it is never simply “stopped” without a plan [8,83,85,88].

### 5.3. Anabolic Agents and Hip-Fracture Prevention: Teriparatide, Abaloparatide, and Romosozumab

#### 5.3.1. Scope and Rationale

Most randomized osteoporosis trials do not report femoral-neck fractures as a standalone endpoint; instead, they prespecify the composite outcome of hip fracture. Accordingly, this section uses hip fracture as the primary trial endpoint and reserves femoral neck for site-specific BMD, structural, or anatomically specific discussions. The most defensible synthesis therefore integrates hip-fracture outcomes with changes at the total hip and femoral neck, as well as sequence-dependent preservation of hip bone mass after the anabolic phase. Recent evidence and expert guidance increasingly support early use of anabolic or dual-acting therapy in patients at very high or imminent fracture risk, because these strategies produce faster skeletal gains and more rapid fracture-risk reduction than an antiresorptive-first approach [9,113,147,148,149].

#### 5.3.2. Teriparatide

Teriparatide remains the best-established parathyroid hormone-based anabolic therapy, but its hip-fracture evidence is indirect compared with vertebral outcomes. The pivotal Neer trial established robust vertebral and non-vertebral fracture efficacy and demonstrated meaningful gains in femoral-neck bone mineral density (BMD), thereby providing the biologic rationale for proximal femoral protection; however, hip-fracture events were too infrequent for a definitive site-specific analysis. In VERO, teriparatide reduced clinical fractures versus risedronate in women with severe postmenopausal osteoporosis, yet the trial was again underpowered for hip fractures. Real-world pooled observational data have nevertheless suggested fewer hip and other fractures during teriparatide treatment in routine practice [90,92,93].

The strongest hip-directed signal for teriparatide comes from meta-analysis. A dedicated systematic review found a significant reduction in hip-fracture odds versus control, and more recent meta-analyses confirm that teriparatide generally outperforms bisphosphonates for overall fracture reduction and improves femoral-neck and total-hip BMD, even if individual randomized controlled trials (RCTs) remain insufficiently powered for hip events [91,150,151].

Treatment sequence is crucial when hip preservation is the dominant clinical objective. Transitioning from teriparatide to a potent antiresorptive consolidates or extends hip gains, whereas switching from denosumab to teriparatide leads to transient loss of hip and forearm BMD and unfavorable microarchitectural changes, making that sequence unattractive when hip-fracture risk is high. Current practice also allows individualized use beyond the historical 24-month ceiling in selected persistently high-risk patients, which is relevant when teriparatide is chosen as the first step in a hip-centered sequential regimen [152,153,154,155].

#### 5.3.3. Abaloparatide

Abaloparatide has a similar conceptual role to teriparatide but a somewhat different evidence profile. In ACTIVE, abaloparatide substantially reduced vertebral and non-vertebral fractures versus placebo, although hip-fracture events were exceptionally rare. Importantly, for proximal femoral protection, ACTIVE-based analyses showed faster and larger BMD response rates at the total hip and femoral neck with abaloparatide than with open-label teriparatide, and ACTIVExtend demonstrated that transition to alendronate preserved and amplified antifracture efficacy after the initial anabolic phase [94,95,97].

Hip-oriented translational evidence for abaloparatide has strengthened in the last few years. ACTIVE-J confirmed gains at the hip in Japanese patients, while a subsequent DXA-based hip structural analysis showed favorable changes in hip geometry and biomechanical indices. A 2024 meta-analysis likewise found significant improvements in femoral-neck and hip BMD, together with vertebral fracture reduction, supporting the view that abaloparatide has meaningful proximal femoral activity even though hip-fracture endpoints remain sparse in the pivotal RCTs [96,98,156].

Comparative evidence has become increasingly interesting. Updated real-world data suggest fewer hip and non-vertebral fractures with abaloparatide than with teriparatide over approximately 18 months, and a 2025 network meta-analysis of PTH1 receptor agonists reached a similar directional conclusion, although the inference is partly informed by observational studies and therefore should not be overinterpreted as equivalent to a hip-powered head-to-head RCT. Abaloparatide also increased BMD in men with osteoporosis, broadening generalizability, and a region-specific proximal femur analysis showed increases in BMD within arthroplasty-relevant Gruen zones, indirectly supporting cortical-site competence. A 2026 systematic review/meta-analysis reinforced this overall pattern of strong BMD efficacy and vertebral protection, while again underscoring the paucity of direct hip-event data [99,100,157,158,159].

#### 5.3.4. Romosozumab

Among currently available bone-forming agents, romosozumab has the clearest direct RCT-level evidence for hip-fracture reduction. In ARCH, one year of romosozumab followed by alendronate significantly reduced hip fractures compared with alendronate alone, alongside reductions in vertebral, clinical, and non-vertebral fractures. By contrast, FRAME was underpowered for hip fracture specifically, but it demonstrated rapid and substantial gains in spine and hip BMD, and the “foundation effect” persisted after transition to denosumab in extension analyses [101,103,104,160].

The hip-centered BMD literature also favors romosozumab. In women transitioning from oral bisphosphonates, STRUCTURE showed greater total-hip BMD gains with romosozumab than with teriparatide over 12 months, and companion imaging analyses documented favorable changes in hip density and mass. Recent meta-analyses have confirmed strong skeletal efficacy overall, while also emphasizing that the possible cardiovascular risk signal remains part of treatment selection and shared decision-making [102,105,106,161].

Recent sequence and real-world studies have made the romosozumab chapter considerably richer than it was even a few years ago. Starting romosozumab after prior bisphosphonate therapy generally produces larger hip and spine gains than switching to denosumab or teriparatide [162,163], and switching from denosumab to romosozumab appears to improve lumbar spine BMD and trabecular bone score more than simply continuing denosumab [164], although hard hip-fracture outcome data are still limited in this setting. Prior teriparatide exposure may attenuate the subsequent romosozumab response [165]. In parallel, contemporary reviews [107] and the Italian ROMEO study [166] support romosozumab as an early, high-impact option in severe osteoporosis, while the 2026 LIDA trial suggests that abbreviated romosozumab followed by denosumab may preserve most of the 12-month total-hip BMD benefit; that latter finding is best viewed as promising sequence evidence rather than definitive fracture-outcome evidence [167].

#### 5.3.5. Comparative Synthesis and Treatment Sequencing

Across contemporary network meta-analyses, romosozumab consistently ranks among the most effective agents for major clinical fracture reduction and is the only anabolic agent with unequivocal direct hip-fracture superiority in a large active-comparator RCT. Teriparatide and abaloparatide are also effective, but the precision of their hip-specific estimates is lower because few individual trials accrued enough hip events. The most reproducible principle across the newer literature is therefore not only agent choice but sequence choice: initiating therapy with an anabolic or dual-acting agent and then consolidating with a potent antiresorptive yields the most dependable preservation of hip BMD and overall fracture benefit [111,112,168,169,170].

From a pragmatic hip-fracture perspective, the agents can be differentiated as follows. Romosozumab has the strongest direct evidence when immediate hip protection is needed and cardiovascular risk is acceptable [103,169]. Teriparatide has meaningful hip-directed support, but that support comes mainly from meta-analysis and integrated observational evidence rather than hip-powered trials [91,151]. Abaloparatide has particularly persuasive femoral-neck and total-hip BMD data, with increasingly favorable indirect and real-world signals versus teriparatide, yet still lacks definitive hip-event RCT confirmation [95,99,100]. For all three agents, the durable strategy is anabolic first, antiresorptive second [97,160,169]; the reverse sequence—especially denosumab followed by teriparatide—should generally be avoided when the dominant clinical threat is hip fracture [152,154].

An important interpretive issue in this section is that the hierarchy of evidence differs across anabolic agents. Romosozumab has the clearest direct hip-fracture evidence [103], whereas the hip-centered case for teriparatide and abaloparatide still relies more heavily on BMD gains, meta-analysis, and observational or sequence data than on hip-powered randomized trials [91,98]. A further unresolved question is how far sequence recommendations can be generalized from surrogate end points to hard hip-fracture outcomes [169,170], especially in very old adults, in men, and in patients with recent antiresorptive exposure or cardiovascular comorbidity [105,106].

#### 5.3.6. Clinical Takeaways for Hip-Centered Prevention

1. Romosozumab has the strongest RCT-level evidence for reducing hip fracture [103] and is the clearest osteoanabolic choice when very rapid hip protection is required [112].
2. Teriparatide remains a valid hip-directed option, but its most convincing support comes from meta-analysis [91] and real-world synthesis [93] rather than from hip-powered randomized trials.
3. Abaloparatide produces robust femoral-neck and total-hip BMD gains [95,98] and may outperform teriparatide in indirect and observational comparisons [99,100], although direct hip-fracture proof remains limited [159].
4. For all anabolic strategies, prompt transition to a potent antiresorptive is essential [97,104,107,152,169] if the aim is sustained hip-fracture prevention.

In summary, a hip-centered reading of the contemporary literature favors an anabolic-first strategy for patients at very high imminent risk of proximal femoral fracture. Within that framework, romosozumab has the strongest direct hip-fracture evidence, teriparatide has the most mature meta-analytic hip signal, and abaloparatide has increasingly persuasive hip-BMD and comparative-effectiveness data that merit serious clinical consideration.

### 5.4. Menopausal Hormone Therapy and SERMs in Primary Prevention of Hip Fractures

#### 5.4.1. Effect of Menopausal Hormone Therapy (MHT) on Fracture Risk

- Menopausal hormone therapy (estrogen alone or combined with progestin) has a well-documented protective effect on bone density and fractures in postmenopausal women [171,172,173,174,175]. Estrogen deficiency after menopause accelerates bone resorption, so restoring estrogen levels with MHT helps maintain bone mass [176]. Large randomized trials, most notably the Women’s Health Initiative (WHI), demonstrated significant reductions in osteoporotic fractures—including hip fractures—among women on MHT (1–3). Because these trials reported composite hip-fracture outcomes rather than isolated intracapsular femoral neck fractures, the evidence in this section should be interpreted as hip-fracture prevention with indirect relevance to femoral neck fracture. In the WHI, 5–7 years of standard-dose conjugated estrogen plus medroxyprogesterone (CEE + MPA) reduced the incidence of hip fractures by roughly one-third compared to placebo (hazard ratio ~0.66) [171]. Estrogen-alone therapy (CEE in women with prior hysterectomy) showed a similar benefit, with about a 28–39% reduction in hip and other fractures [172,173]. A 2017 meta-analysis of long-term hormone therapy confirmed that fracture risk was the one major outcome with robust evidence of benefit, with conventional-dose MHT significantly lowering vertebral and hip fractures in postmenopausal women [174,175]. These bone benefits are observed in women both with and without established osteoporosis, and even those with osteopenia gain protection from fractures while on therapy [171,172,173,174,175,177]. Notably, the bone-protective effect is dose-dependent (higher doses yield greater BMD gains) and dissipates after discontinuing therapy—fracture rates begin to rise back to baseline within a few years of stopping MHT [178,179,180,181,182]. This was seen in follow-ups of WHI: women lost the fracture protection once hormones were stopped, especially if therapy duration was under 5 years [180,181,182,183,184,185]. More precisely, post-discontinuation loss of fracture protection is supported more directly by cessation studies such as NORA and Karim et al., whereas WHI post-intervention follow-up mainly showed attenuation of on-treatment benefit rather than a uniformly demonstrated rebound versus former placebo.

Current guidelines consider MHT an appropriate option for primary prevention of bone loss and fractures in younger postmenopausal women (typically <60 years or within 10 years of menopause) who are at elevated fracture risk and have no contraindications [186,187]. The North American Menopause Society, for example, states that hormone therapy has been shown to prevent postmenopausal bone loss and osteoporotic fractures (spine and hip) and has a favorable benefit–risk profile for women under 60 or within 10 years of menopause when other risk factors are absent [186]. However, for older women beyond their mid-60 s, or those more than 10 years menopausal, the balance shifts—non-hormonal osteoporosis medications are usually preferred, since long-term MHT in older age carries greater health risks [186,187,188].

Evidence from RCTs: The fracture-prevention efficacy of MHT was first conclusively shown in WHI. In WHI’s estrogen + progestin trial (~16,000 women), hip fracture incidence was reduced by about 33–35% in the hormone group [189]. Similarly, the estrogen-alone WHI trial (~10,000 women) showed a ~30% reduction in total osteoporotic fractures (HR 0.72, 95%CI 0.64–0.80) [190]. These findings are supported by earlier trials and observational studies. For example, the National Osteoporosis Risk Assessment (NORA) study (over 200,000 postmenopausal women) found that current hormone therapy users had significantly fewer fractures (including hip, spine, wrist, and rib) than non-users [189]. In that cohort, the fracture risk reduction disappeared after ~5 years off MHT, emphasizing the need for ongoing therapy to maintain benefit [189]. A Cochrane review of long-term HT likewise concluded that women on estrogen (with or without progestin) have lower rates of vertebral and non-vertebral fractures, while women who never took HT or who stopped it lose that advantage [189].

Magnitude of Benefit: In practical terms, standard-dose MHT produces improvements in bone mineral density (~3–5% gains at the hip and spine over 3 years) and approximately a one-third reduction in fracture risk at major sites [189]. This antifracture effect of estrogen is comparable to that of first-line osteoporosis drugs in early postmenopausal women. It is important to note MHT’s effect is preventive—it is most effective when initiated around the time of menopause, rather than in very elderly women. MHT is not typically used as the sole therapy in women over 60 purely to prevent fractures, because of age-related risks, but it can be a valuable primary prevention strategy in younger menopausal women who need menopausal symptom relief and also have risk factors for hip fracture [189].

#### 5.4.2. Effects of SERMs on Fracture Prevention

Selective estrogen receptor modulators (SERMs) are oral agents that selectively mimic or block estrogen’s action in various tissues. Several SERMs have been used to prevent or treat osteoporosis, aiming to confer estrogen-like benefits on bone without stimulating breast or uterine tissue. The SERMs most relevant to bone health include raloxifene, bazedoxifene, and lasofoxifene (tamoxifen is also a SERM but is primarily used for breast cancer prevention, not osteoporosis). These agents reduce bone turnover and preserve BMD, especially at the spine, and they significantly cut vertebral fracture incidence. However, their ability to prevent hip fractures is limited, and isolated femoral neck-fracture data are generally not available because clinical trials have not reported that endpoint separately [191].

- Raloxifene: Raloxifene is a second-generation SERM that has estrogen-agonist effects in bone and lipid metabolism but antagonistic effects in breast and uterine tissue. It is FDA-approved for osteoporosis prevention and treatment in postmenopausal women. The landmark Multiple Outcomes of Raloxifene Evaluation (MORE) trial (7705 osteoporotic women, 4-year duration) demonstrated that raloxifene 60 mg/day increases BMD and reduces vertebral fractures by ~30–50% (depending on baseline fracture status) [191,192]. Specifically, women on raloxifene had about a 40–50% lower risk of new spinal fractures versus placebo. Crucially, though, MORE and subsequent studies found no significant reduction in hip or other non-vertebral fractures with raloxifene [191]. Incidence of hip fractures was low and did not differ from placebo, indicating that raloxifene’s antifracture efficacy is largely confined to the spine. A pooled analysis confirmed that while raloxifene consistently lowers vertebral fracture risk, it does not significantly decrease hip fracture risk in postmenopausal women [191]. Raloxifene’s clinical role has therefore been focused on women at high risk of vertebral fracture (e.g., those with spinal osteoporosis), especially if they also could benefit from raloxifene’s extra advantage of reducing breast cancer risk. In MORE and its extension study, raloxifene use was associated with a 70% reduction in invasive estrogen-receptor positive breast cancer compared to placebo. This unique dual benefit (spine protection and breast cancer risk reduction) makes raloxifene an attractive option for younger postmenopausal women with osteopenia/osteoporosis who also have elevated breast cancer risk. However, for femoral neck (hip) fracture prevention, raloxifene is not as effective as other osteoporosis medications (like bisphosphonates or estrogen); as no RCT to date has demonstrated a statistically significant reduction in hip fracture risk [191].
- Bazedoxifene: Bazedoxifene is a third-generation SERM that was tested in large Phase III trials for osteoporosis. In a 3-year trial of 7492 high-risk postmenopausal women, bazedoxifene (20 mg or 40 mg daily) significantly reduced the incidence of new vertebral fractures by ~40% versus placebo [191]. The vertebral fracture risk was reduced both at 20 mg (HR ~0.58) and 40 mg (HR ~0.63) doses [193]. The effect on non-vertebral fractures was more modest: in the overall study population, bazedoxifene did not significantly reduce non-vertebral fractures compared to placebo [194,195]. However, in a higher-risk subgroup of women (e.g., those with severe osteoporosis or prior fractures), bazedoxifene showed a relative reduction in non-vertebral fractures (~50% vs. placebo)—a benefit that approached significance in post hoc analyses [196]. Overall, no definitive reduction in hip fracture risk was demonstrated in the primary bazedoxifene trials, likely due to the low incidence of hip fractures in the study cohorts and insufficient power to detect differences [191,194]. Despite this, bazedoxifene effectively increases BMD at both spine and hip [191], and a 2017 meta-analysis of four RCTs confirmed that bazedoxifene significantly lowers vertebral fracture risk and is well tolerated over up to 7 years of use. Bazedoxifene is not available as a standalone osteoporosis drug in some countries; instead, it has been combined with conjugated estrogens as a tissue-selective estrogen complex (TSEC). The conjugated estrogen/bazedoxifene combination (brand name Duavee) is approved for use in postmenopausal women with a uterus to treat vasomotor symptoms and prevent osteoporosis. Clinical trials of this CE + BZA combination have shown it maintains BMD and prevents bone loss, comparable to estrogen therapy, while also protecting the endometrium (bazedoxifene acts as the progestogen substitute) [191]. This TSEC offers an alternative for early postmenopausal women who need estrogen for symptom relief but cannot take a progestin—it provides fracture prevention similar to estrogen alone, though long-term fracture outcomes (especially hip fractures) with TSEC have not been extensively studied in outcomes trials [191].
- Lasofoxifene: Lasofoxifene is another third-generation SERM investigated for osteoporosis [197]. The Phase III PEARL trial (Postmenopausal Evaluation and Risk Reduction with Lasofoxifene) followed 8556 women (ages 59–80, all with low BMD and/or existing vertebral fractures) for 5 years [198]. Lasofoxifene 0.5 mg/day produced a significant 42% reduction in vertebral fractures compared to placebo (13.1 vs. 22.4 cases per 1000 person-years) and a 24% reduction in non-vertebral fractures (hazard ratio ~0.76) [198]. This broader antifracture effect was something not observed with raloxifene—indeed, the trial authors highlighted that lasofoxifene reduced non-spine fractures, whereas raloxifene had not in prior studies [198,199]. However, it is important to contextualize the non-vertebral fracture finding: the reduction in non-vertebral fractures with lasofoxifene was driven mostly by fewer wrist and ankle fractures [199]. No statistically significant decrease in hip fracture incidence was reported with lasofoxifene, likely because hip fractures were relatively infrequent in the trial (the population’s average age was around 67 and many were younger than the typical hip fracture demographic) [198,199]. An editorial accompanying the PEARL study noted that the absolute risk reduction for non-vertebral fractures was small and became apparent only after 5 years of therapy [199]. Thus, while lasofoxifene may have a slight advantage over raloxifene in non-vertebral fracture efficacy, neither SERM has demonstrated a robust reduction in hip fractures in an RCT [198,199]. Lasofoxifene did show intriguing extra-skeletal benefits: in PEARL, the 0.5 mg dose reduced ER-positive breast cancer risk by 81% (similar to raloxifene’s breast protective effect) and unexpectedly reduced the risk of coronary events by ~32% and stroke by ~36% [198,200,201,202]. These findings suggest lasofoxifene might confer multisystem benefits, but they come with the caveat of SERM class side effects [198,201]. Lasofoxifene has not been widely approved for osteoporosis, but it remains of interest (it is being studied for breast cancer treatment in certain settings, leveraging its ER antagonist activity in breast tissue) [197,203].

In summary, SERMs provide significant protection against vertebral fractures, but their ability to prevent hip fractures is unproven or minimal. Head-to-head comparisons suggest SERMs are less potent than bisphosphonates or estrogen for hip fracture prevention. For example, raloxifene failed to reduce hip fractures in trials [191], and bazedoxifene’s and lasofoxifene’s benefits for hip fractures were at best marginal. Therefore, in a postmenopausal woman at high risk of hip fracture, a SERM alone would not be the first-line choice for fracture prevention. Instead, SERMs are typically used in women at lower absolute hip fracture risk but who seek vertebral fracture protection—often younger postmenopausal women or those who cannot tolerate other osteoporosis drugs. A particular niche is the woman with osteoporosis or osteopenia who is also at increased risk of breast cancer: for her, a SERM like raloxifene or lasofoxifene could simultaneously address both concerns (bone and breast).

#### 5.4.3. Safety Considerations: MHT vs. SERMs

When evaluating MHT or SERMs for fracture prevention, their safety profiles and extra-skeletal effects must be considered.

Menopausal Hormone Therapy Risks: MHT’s benefits on bone (and perhaps colon cancer and menopausal symptoms) must be weighed against its potential harms. The WHI and other studies found that combined estrogen–progestin therapy (EPT) is associated with increased risks of breast cancer, coronary heart disease, stroke, venous thromboembolism (VTE), and gallbladder disease, especially when used in older women [190]. Estrogen-alone therapy (ET) has a different risk profile: it does not increase breast cancer risk (in fact, WHI found estrogen-alone slightly lowered breast cancer incidence in women without a uterus), but it still carries elevated risks of stroke, VTE, and ovarian cancer [190]. Both EPT and ET can increase the risk of ischemic stroke and blood clots (deep vein thrombosis/pulmonary embolism), with risk magnitude depending on age and route of administration (oral estrogen confers higher thrombotic risk than transdermal) [190]. Importantly, the absolute risk of these events in the 50–59 age group is low. Current guidelines advise using the lowest effective dose for the shortest duration consistent with the patient’s goals and to individualize therapy. For a healthy 50-year-old menopausal woman with significant fracture risk, a carefully monitored course of MHT may yield a favorable risk–benefit balance [186,189]. But in an older woman (e.g., >65) purely seeking fracture prevention, the same therapy could incur disproportionate risks. Long-term MHT is not recommended solely for fracture prevention in women over 60 [189]; other osteoporosis-specific medications would usually be preferred in that scenario.

It is also noteworthy that if MHT is discontinued, bone loss accelerates and fracture risk increases back toward baseline, as discussed. Women who stop hormone therapy should be evaluated for alternative bone-protective strategies to avoid rapid decline in BMD and heightened hip fracture risk in the years following discontinuation.

SERM Risks: SERMs do not carry the risk of breast or endometrial cancer that hormones can (raloxifene and bazedoxifene actually reduce estrogen-sensitive breast cancer risk, and they do not stimulate the endometrium). However, SERMs share some of estrogen’s side effects and risks. A class effect of SERMs is an increased risk of venous thromboembolism—similar in magnitude to estrogen. Raloxifene approximately doubles the risk of deep vein thrombosis and pulmonary embolism; in the MORE and RUTH trials, thromboembolic event rates were higher with raloxifene than placebo (RR ~2–3) [191]. Lasofoxifene in the PEARL trial likewise more than doubled the relative risk of VTE compared to placebo (although absolute incidence was low). Due to this, SERMs are contraindicated in women with active or past thromboembolic events and should be used cautiously in those with significant risk factors for VTE (such as prolonged immobilization) [191]. Another common SERM side effect is vasomotor symptoms—paradoxically, SERMs can cause or worsen hot flashes. Raloxifene often induces hot flashes and leg cramps in a subset of women, which can limit tolerability [191]. Bazedoxifene when given alone can cause hot flashes as well; interestingly, when bazedoxifene is combined with conjugated estrogen (in a TSEC), it blunts estrogen’s stimulatory effect on the breast/uterus but allows the estrogen to alleviate hot flashes, so the combination does not cause hot flashes the way bazedoxifene alone might.

Cardiovascular effects of SERMs have been mixed. Unlike estrogen, SERMs do not appear to confer cardioprotection. In the Raloxifene Use for The Heart (RUTH) trial (10,000 postmenopausal women with high CV risk), raloxifene showed no significant reduction in coronary heart disease events compared to placebo [191]. There was, however, a slight increase in fatal stroke observed with raloxifene in women with risk factors for stroke [191]. This suggests that in older women with a high baseline stroke risk, raloxifene might exacerbate that risk (one hypothesis is that SERMs, like estrogen, can increase coagulation and thus thrombotic stroke risk in susceptible individuals) [191]. Lasofoxifene’s trial hinted at fewer overall strokes and cardiac events, but those findings need confirmation and may not translate to other populations. Neither raloxifene nor lasofoxifene had any adverse effect on endometrial health—in fact, bazedoxifene has demonstrated anti-estrogenic effects on the uterus, even reducing endometrial thickness. This is a safety advantage over tamoxifen (a SERM used in breast cancer), which is known to increase endometrial cancer risk; raloxifene and bazedoxifene do not increase endometrial cancer.

Overall, for primary prevention of hip fractures, MHT has the stronger evidence of efficacy, whereas femoral neck-specific benefit is inferred from the composite hip-fracture endpoint rather than separately demonstrated. Its use is tempered by broader systemic risks. SERMs offer a partial alternative—they are safer with respect to breast/uterine cancer and can be used long-term for osteoporosis, but they do not match estrogen or other osteoporosis drugs in hip-fracture reduction. The choice often comes down to an individualized risk–benefit assessment. For a younger postmenopausal woman with moderate fracture risk and severe menopausal symptoms, systemic hormone therapy can both improve quality of life and prevent bone loss (with hip-fracture risk reduction) [186,189]. In contrast, a woman who cannot take estrogen (e.g., due to a history of breast cancer or high breast cancer risk) might be offered a SERM like raloxifene, accepting that it will primarily protect against vertebral fractures and provide some extra skeletal benefits (less breast cancer, improved lipid profile) but with careful monitoring for VTE. In all cases, adequate calcium/vitamin D, lifestyle measures (exercise, fall prevention), and periodic re-evaluation of therapy are recommended alongside these interventions.

A critical distinction is that skeletal efficacy alone does not determine clinical place. For MHT, the controversy is not whether hip-fracture reduction exists [171,174] but in which women the skeletal benefit outweighs age-dependent risks of breast cancer, thromboembolism, stroke, and other systemic harms [186,190]. For SERMs, the unresolved issue is almost the reverse: they offer a favorable vertebral and breast-risk profile in selected women, yet their limited and largely indirect hip-fracture evidence makes extrapolation to hip-centered prevention clinically weak [191,198].

#### 5.4.4. Summary

Menopausal hormone therapy is effective in preventing osteoporosis-related fractures, including hip fractures, in postmenopausal women. RCT evidence (WHI and others) shows roughly a 30–40% reduction in hip-fracture risk during active treatment [189]. Because the available trial evidence is reported as hip fracture rather than isolated femoral neck fracture, femoral-neck-specific benefit is inferred rather than separately demonstrated. However, this benefit must be balanced against the known risks of long-term hormone therapy, and current practice reserves MHT for women who also need menopausal symptom relief or who are at low risk for its adverse effects. SERMs such as raloxifene, bazedoxifene, and lasofoxifene also play a role in primary prevention of fractures. They significantly reduce vertebral fracture incidence [191] and maintain bone density, but they have not conclusively been shown to prevent hip fractures to the same extent. Their use is appropriate in women with predominant spine osteoporosis or those who cannot use other therapies, especially when there is a concurrent need for breast cancer risk reduction. Combining a SERM with estrogen (as in CE + bazedoxifene) is an emerging strategy that can protect bone and treat menopausal symptoms without progestins. Ultimately, preventing hip fractures in postmenopausal women may require a multifaceted approach: risk stratification, lifestyle optimization, and pharmacotherapy tailored to the individual’s risk profile and treatment goals. Both MHT and SERMs have evidence-based roles in this spectrum, with MHT offering the greater fracture risk reduction (including at the hip) [189], and SERMs offering a targeted option for vertebral fracture prevention with a different side-effect profile. Ongoing research and newer agents (e.g., tissue-selective estrogen complexes, or other novel SERMs) continue to refine our ability to protect skeletal health while minimizing risks for postmenopausal women. A hip-oriented comparison of hormonal and adjunctive agents is provided in Table 2. The key trials and evidence syntheses supporting this review are listed in Table 3.

## 6. Secondary Prevention and Health-System Interventions

Secondary prevention after an initial hip fracture is one of the highest-yield opportunities in osteoporosis care. A hip fragility fracture should be regarded as a sentinel event that automatically triggers skeletal evaluation, falls assessment, and anti-osteoporosis treatment, rather than as an isolated orthopedic episode. Contemporary consensus recommendations support pharmacologic secondary prevention after an initial fragility fracture of the hip, vertebra, or pelvis regardless of bone mineral density. However, real-world care remains inconsistent: in a multinational analysis, post-hip-fracture treatment rates ranged from 11.5% to 50.3% across health systems, and in a contemporary population-based cohort fewer than one-third of older adults filled an anti-osteoporosis prescription within 1 year after discharge [2,10,204].

### 6.1. Fracture Liaison Services

Fracture liaison services (FLSs) are the best-studied health-system intervention for closing this care gap. The core FLS model is coordinator-based and includes systematic case findings after fragility fracture, risk assessment, bone-health investigation, initiation of evidence-based therapy, communication with primary care, and support for long-term persistence. The IOF Capture the Fracture framework and the subsequent international patient-level KPI set have provided a practical structure for implementation and audit, including metrics for identification, time to assessment, treatment initiation, and follow-up [205,206]. Evidence syntheses consistently show that FLS programs improve bone mineral density testing, treatment initiation, and treatment adherence, and the most recent systematic review and meta-analysis found moderate-certainty evidence that FLS reduces secondary fragility fractures when follow-up extends to 2 years or longer [11,207,208].

### 6.2. Orthogeriatric Co-Management

Orthogeriatric co-management complements FLS by addressing the acute medical and geriatric complexity of hip-fracture patients. Population-based and meta-analytic evidence indicates that orthogeriatric care is associated with lower in-hospital and longer-term mortality, shorter length of stay, less delirium, and better care processes, while also improving the likelihood of osteoporosis evaluation and treatment [209,210,211,212]. A large multicenter non-randomized study from China further showed that co-management pathways can shorten time to surgery, improve clinical management, and reduce 1-year mortality [213]. Importantly, no single orthogeriatric configuration has been proved universally superior, but the overall evidence favors routine integrated geriatric involvement over ad hoc consultation alone [210,211].

### 6.3. Rehabilitation and Early Mobilization

Secondary prevention after hip fracture also depends on structured recovery pathways. Multidisciplinary rehabilitation probably reduces mortality and may improve function and mobility, while early mobilization after surgery is associated with lower 30-day mortality and fewer complications [214,215]. In practice, this means that secondary prevention pathways should extend beyond surgery and drug prescription to include early mobilization, progressive rehabilitation, reassessment of gait and balance, medication review, nutritional optimization, and linkage to community falls-prevention services after discharge [10,214,215].

### 6.4. Early Pharmacologic Secondary Prevention

The pharmacologic component of secondary prevention should be organized as a default pathway rather than deferred to uncertain outpatient follow-up. In the HORIZON Recurrent Fracture Trial, zoledronic acid initiated after surgical repair of a recent hip fracture reduced new clinical fractures and improved survival [82]. More recent implementation studies indicate that starting osteoporosis treatment during the index hospitalization increases the likelihood that treatment is actually delivered. Inpatient zoledronic acid improved treatment rates, surgeon-led in-hospital algorithms improved persistence and may help prevent second fragility fractures, and standardized hip-fracture order sets increased treatment initiation and 1-year persistence even when reductions in secondary fracture were not yet demonstrable [216,217,218]. These findings are especially important in very old adults, for whom missed follow-up after discharge is common.

### 6.5. System-Level Implementation Priorities

From a health-system perspective, the key lesson is that secondary fracture prevention succeeds when responsibility is explicit. Hip-fracture pathways should therefore include automatic case finding, orthogeriatric review, falls-risk assessment, basic bone-health optimization, in-hospital or very early post-discharge initiation of anti-osteoporosis therapy, and clear transfer of long-term follow-up to FLS or primary care. KPI-driven audit is essential [10,205,206]. In a recent population-based study, higher adherence to hip-fracture clinical care standards was associated with substantially lower 30-day and 365-day mortality; orthogeriatric care and mobilization by the day after surgery were independently associated with better survival [219].

Although the health-system signal is persuasive [207,208,209], the evidence base is methodologically more heterogeneous than guideline summaries sometimes imply. Many studies of FLS and orthogeriatric care are observational, use different service configurations, or emphasize process measures such as testing and treatment initiation more than second-hip-fracture outcomes [207,208,209,216,217,218], so the specific components responsible for benefit remain difficult to isolate. An important unresolved question is therefore not whether coordinated care matters but which combination of inpatient initiation [216,217,218], geriatric co-management [209], rehabilitation [214,215], adherence support, and long-term follow-up [205,206,207,208] is necessary to produce reproducible reductions in recurrent hip fractures across different resource settings.

In our view, secondary-prevention pathways fail when responsibility is diffuse. Each hip-fracture pathway should therefore designate a named accountable owner for post-fracture case findings, treatment initiation, follow-up completion, and audit. Where an FLS is available, this role is most naturally fulfilled by the FLS coordinator or service. Where FLS is not available, accountability should default to the orthogeriatric or hip-fracture service during admission, with explicit written handover to a named outpatient clinician or primary-care team after discharge.

### 6.6. Clinical Implications

For clinicians, the practical implication is straightforward: secondary prevention after hip fracture is primarily a systems problem, not a knowledge problem. Effective drugs already exist, but they must be delivered through reliable coordinated pathways. The strongest contemporary model is one in which a hip fracture automatically triggers orthogeriatric co-management, multidisciplinary rehabilitation, prompt anti-osteoporosis treatment, falls assessment, and monitored follow-up through FLS or an equivalent coordinated service. When implemented consistently, such pathways can narrow the osteoporosis care gap and reduce recurrent fragility fractures, including second hip fractures [10,11,82,205,219].

The most transferable examples in the current literature are not isolated educational initiatives but default pathways: coordinator-based FLS, orthogeriatric co-management, inpatient treatment initiation, standardized order sets, and KPI-based audit. In practical terms, the minimum deliverable model across health systems is automatic case findings after hip fracture, in-hospital or immediate post-discharge osteoporosis treatment, documented falls-risk assessment, a named follow-up provider, and audit of treatment completion and persistence. High-resource systems may deliver this through integrated FLS and orthogeriatrics, whereas lower-resource systems may rely on surgeon-led or hospitalist-led pathways linked to primary care, but the essential feature is the same—accountability must be assigned before the patient leaves the hospital.

### 6.7. Summary

In summary, secondary prevention after hip fracture should be organized as a default system pathway rather than left to opportunistic outpatient follow-up. The most effective model combines automatic post-fracture case findings, fracture liaison services, orthogeriatric co-management, early rehabilitation and mobilization, prompt initiation of anti-osteoporosis therapy, falls assessment, and accountable long-term follow-up. The consistent message across this section is that recurrent hip-fracture prevention is primarily an implementation challenge: effective treatments already exist, but they reduce second-fracture burden only when delivered through coordinated, audited care pathways that define responsibility across hospital and community settings.

## 7. Conclusions

Preventing hip fractures requires a broader perspective than osteoporosis treatment alone. In the literature reviewed here, hip fracture is the principal reported clinical endpoint, whereas femoral neck fracture is discussed when anatomically specific data or femoral-neck BMD measures are available. Hip-fracture risk arises from the convergence of skeletal fragility, falls, sarcopenia, frailty, multimorbidity, and aging-related functional decline. Although age-standardized incidence has declined in several settings, the absolute burden continues to increase with population aging. The evidence reviewed here indicates that no single intervention is sufficient; the most effective strategy is integrated, risk-stratified, and sustained over time.

For primary prevention, exercise-based fall prevention remains fundamental, particularly programs that challenge balance and improve lower-extremity strength. Calcium and vitamin D appear most useful in selected patients with deficiency, low intake, or institutional residence, whereas hip protectors are best reserved for frail residents of long-term care settings. Pharmacologic therapy is central for patients with osteoporosis or high fracture probability. Bisphosphonates and denosumab have the strongest antiresorptive evidence for hip-fracture reduction, while anabolic-first approaches, especially romosozumab-based sequencing, may be preferable in patients at very high or imminent risk.

A consistent message across this review is that the major remaining barrier is implementation. Effective therapies and preventive strategies already exist, yet case findings, treatment initiation, persistence, and post-fracture follow-up remain inadequate in routine practice. Fracture liaison services, orthogeriatric co-management, early treatment after fragility fracture, and adherence support should therefore be regarded as core components of prevention, not optional additions.

Ultimately, meaningful reductions in hip-fracture burden will depend on combining skeletal protection, falls prevention, and coordinated health-system delivery. This integrated model offers the most realistic path to reducing both first and recurrent hip fractures in aging populations. From a pragmatic clinician-oriented perspective, prophylaxis after an index hip fracture should ideally begin before hospital discharge, under the responsibility of the orthogeriatric or hip-fracture team, or through an FLS where available, with explicit handover for long-term follow-up. For most patients, this means prompt initiation of a hip-fracture-effective anti-osteoporosis therapy, combined with rehabilitation, falls-risk assessment, and correction of calcium or vitamin D deficiency when present. In patients with osteoporosis but no prior hip fracture, management should remain risk-stratified: exercise and falls prevention for all, bisphosphonates as first-line therapy for many high-risk patients, denosumab when oral adherence or bisphosphonate suitability is problematic, and anabolic-first strategies for those at very high or imminent fracture risk. A clinician-oriented summary of these interventions is provided in Table 4.

## Figures and Tables

**Table 1:** Antiresorptive and osteoanabolic therapies: representative hip/femoral-neck BMD effects and fracture outcomes relevant to proximal femur prevention.

Agent/Class	Representative Hip/FN BMD Effect	Fracture Reduction Profile	Hip-Fracture/Proximal-Femur Relevance	Hip-Centered Interpretation
Alendronate	Consistent hip BMD increase during treatment [76,77].	Clear vertebral and broader clinical fracture reduction in high-risk osteoporosis [76,77].	FIT vertebral-fracture cohort: hip fracture reduced by about 51% over 3 years [76]; in lower-risk women without vertebral fracture the overall hip-fracture reduction was not statistically significant, although the osteoporotic subgroup benefited [77,78].	Good first-line oral option when secondary prevention or clearly osteoporotic hip BMD is present [76,77,78].
Risedronate	Modest but clinically meaningful hip/femoral-neck BMD increase [79].	Reduces vertebral and non-vertebral fractures in osteoporotic populations [78,79].	HIP trial: hip fracture reduced by about 40% in women aged 70–79 years with osteoporosis [79]; benefit was not clear in women aged 80 years or older enrolled mainly on non-skeletal risk factors [79].	Most persuasive when osteoporosis—rather than age/falls risk alone—drives treatment selection [78,79].
Zoledronic acid	Potent and durable hip BMD gain with once-yearly intravenous dosing [80,81].	Substantial vertebral and non-vertebral fracture reduction [80,82].	HORIZON-PFT: hip fracture reduced by 41% over 3 years [80]; after surgical hip-fracture repair, new clinical fractures fell by 35% and mortality by 28% [82].	Strong intravenous choice for both primary and secondary proximal-femur prevention, especially when adherence is a concern [78,80,82].
Denosumab	Progressive total-hip/femoral-neck BMD gain; achieved hip T-score predicts lower later fracture risk [83,84,85]; hip strength also improves [86].	Robust vertebral and non-vertebral fracture reduction in placebo-controlled RCTs [83,87].	FREEDOM: hip fracture reduced by about 40% versus placebo [83]; the absolute benefit was larger in women aged 75 years or older and in those with low baseline femoral-neck BMD [87].	Excellent option when oral adherence is poor or bisphosphonates are unsuitable, but on-time dosing and a sequential stop plan are essential [88,89].
Teriparatide	Increases femoral-neck and hip BMD, although gains are generally smaller than at the spine [90,91].	Strong vertebral efficacy and lower clinical-fracture risk versus risedronate in severe osteoporosis [90,92].	Direct hip-powered RCT evidence is limited; support for hip protection comes mainly from meta-analysis and integrated real-world analyses showing fewer hip and upper-limb fractures [91,93].	Valid anabolic option when hip preservation is desired, but hip-fracture evidence is less direct than for romosozumab [91,93].
Abaloparatide	Rapid total-hip/femoral-neck BMD gain; hip response rates exceed placebo and are faster than with teriparatide in ACTIVE analyses [94,95,96].	Strong vertebral and non-vertebral fracture reduction during the abaloparatide-to-alendronate sequence [94,97].	Direct hip-fracture RCT evidence remains limited because hip events were rare, but recent meta-analyses and real-world comparative data are directionally favorable [98,99,100].	Useful anabolic option when followed by antiresorptive consolidation; direct hip-fracture proof remains less definitive than for romosozumab [97,98,100].
Romosozumab	Rapid and large hip BMD gain over 12 months; total-hip gains exceed those seen with teriparatide after prior bisphosphonate therapy [101,102].	Reduces vertebral, clinical, and non-vertebral fractures [101,103,104].	ARCH provides the clearest active-comparator RCT evidence, with fewer hip fractures after romosozumab followed by alendronate than with alendronate alone [103]; FRAME supported rapid hip BMD improvement but was not hip powered [101,104].	Best osteoanabolic/dual-acting option when very rapid hip protection is needed and cardiovascular risk is acceptable; follow with potent antiresorptive therapy [103,105,106,107].

**Table 2:** Hormonal and adjunctive agents: hip-oriented interpretation of BMD and fracture outcomes.

Agent/Class	Representative Hip/FN BMD Effect	Fracture Reduction Profile	Hip-Fracture/Proximal-Femur Relevance	Hip-Centered Interpretation
Menopausal hormone therapy (MHT)	Prevents bone loss and increases hip BMD while therapy is continued [171,173].	Broad fracture reduction during active treatment [171,173].	The WHI estrogen-plus-progestin and estrogen-alone trials both showed reduced hip-fracture risk [171,173].	Bone efficacy is real, but MHT is usually selected for younger symptomatic postmenopausal women with favorable overall risk profiles, not as stand-alone osteoporosis therapy for older high-risk women [186,188].
SERMs (raloxifene/bazedoxifene/lasofoxifene)	Modest preservation of or increase in hip BMD; spine effects are generally greater than hip effects [192,194].	Consistent vertebral-fracture reduction [192,194]; lasofoxifene also reduced non-vertebral fractures in PEARL [198].	Robust hip-fracture reduction is not established for raloxifene or bazedoxifene [192,194]; lasofoxifene remains interesting but still lacks the level of direct hip-fracture proof seen with major antiresorptives or romosozumab [111,112,198].	Useful when vertebral protection and/or breast-risk reduction is a priority, but not first-line when hip fracture is the dominant endpoint [112,192,194,198].
Calcium + vitamin D	Attenuates femoral-neck bone loss and corrects secondary hyperparathyroidism when baseline intake is low [67,68].	The clearest benefit is seen in frail institutionalized older adults [67,70].	Decalyos I showed hip fracture reduced by 43% over 18 months [67]; community-dwelling results are smaller, mixed, and adherence-dependent [70,71,72].	Best viewed as adjunctive and setting-specific, not a stand-alone substitute for proven osteoporosis drugs in most community dwellers [70,72].
Vitamin D alone	Minimal or inconsistent skeletal effect in vitamin-D-replete community adults [58,60].	No meaningful fracture reduction in large modern RCTs or meta-analyses [58,59,60].	VITAL showed no hip-fracture reduction [59]; annual high-dose bolus dosing increased falls and fractures in older women [61]; a recent meta-analysis in healthy elderly populations was also unsupportive [64].	Use to treat deficiency, not as stand-alone hip-fracture prophylaxis in replete community-dwelling adults [58,60,64].
Calcium alone	Only a small hip BMD gain (about 1%) that plateaus early [66].	No convincing reduction in hip fracture [65].	Insufficient as a stand-alone strategy for proximal-femur fracture prevention [65,66].	Reasonable only to correct low intake; otherwise, it is supportive background care rather than definitive fracture-prevention therapy [65,66].

**Table 3:** Anchor trials and syntheses for the narrative text.

Theme/Agent	Landmark Study/Synthesis	Suggested Manuscript-Ready Statement	Use in the Review
High-risk oral bisphosphonate therapy	FIT vertebral-fracture cohort [76]; FIT low-BMD/no-vertebral-fracture cohort [77].	Alendronate has direct RCT evidence for hip-fracture reduction in women with established osteoporosis/high baseline risk, whereas the signal is weaker in lower-risk women without prior vertebral fracture [76,77].	Good anchor when discussing risk-stratified efficacy within oral bisphosphonates.
Elderly osteoporotic women	HIP trial [79].	Risedronate reduces hip fractures when osteoporosis is documented, but age/falls risk alone is an inadequate proxy for skeletal fragility [79].	Useful for explaining why BMD-defined risk matters in the oldest old.
Intravenous bisphosphonate/secondary prevention	HORIZON-PFT [80]; HORIZON-RFT [82].	Zoledronic acid provides some of the strongest hip-centered evidence among bisphosphonates, with both primary hip-fracture reduction and lower subsequent fracture burden after a sentinel hip fracture [80,82].	Strong anchor for both primary and secondary prevention sections.
Denosumab	FREEDOM [83]; high-risk subgroup analysis [87]; discontinuation literature [88,89].	Denosumab has direct placebo-controlled evidence for hip-fracture reduction, but its effectiveness in practice depends on on-time dosing and planned sequential therapy at discontinuation [84,87,88,89].	Good anchor for efficacy plus implementation caveats.
Teriparatide	Neer trial [90]; VERO [92]; meta-analysis [91].	For teriparatide, hip protection is supported mainly by indirect convergence of BMD, meta-analytic, and real-world data rather than by a hip-powered randomized trial [90,91,92,93].	Helps distinguish strong biologic plausibility from direct hip-trial proof.
Abaloparatide	ACTIVE [94]; ACTIVExtend [97]; recent meta-analysis/RWE [98,99,100].	Abaloparatide produces rapid hip BMD gains and strong vertebral/non-vertebral efficacy, but direct hip-fracture evidence remains limited because hip events were rare in pivotal trials [94,95,97,98].	Useful when emphasizing hip-BMD responsiveness and the need for sequence interpretation.
Romosozumab	ARCH [103]; FRAME/extension [101,104]; STRUCTURE [102].	Romosozumab is the clearest osteoanabolic option for direct hip-fracture prevention because ARCH showed significantly fewer hip fractures than alendronate alone, while FRAME and STRUCTURE support rapid hip-BMD improvement [101,102,103,104].	Best anchor for an anabolic-first strategy when immediate hip protection matters.
Hormonal therapy and SERMs	WHI fracture analyses [171,173]; raloxifene/bazedoxifene/lasofoxifene trials [111,192,194,198].	MHT reduces hip fractures during active treatment, whereas SERMs are better viewed as vertebral-focused therapies with less convincing hip-fracture protection [171,173,192,194,198].	Helps position MHT and SERMs within a hip-centered pharmacologic hierarchy.
Vitamin D/calcium	Decalyos I/II [67,68]; VITAL [59]; umbrella/systematic reviews [58,70,72].	Calcium plus vitamin D has its clearest hip-fracture benefit in frail institutionalized older adults with low intake, whereas vitamin D alone does not prevent hip fractures in replete community-dwelling adults [58,59,67,68,72].	Useful for separating supportive supplementation from definitive anti-osteoporosis drug therapy.
Overarching syntheses	Network meta-analyses and ACP living review/update [9,111,112,147].	Across recent syntheses, bisphosphonates and denosumab consistently reduce hip fractures versus placebo, while anabolic therapies generally rank highest for broader clinical-fracture reduction in very-high-risk patients [9,111,112,147].	Best umbrella citation for comparative interpretation across classes.

Abbreviations: BMD = bone mineral density; RCT = randomized controlled trial; MHT = menopausal hormone therapy; SERM = selective estrogen receptor modulator. BMD statements are representative rather than strictly cross-trial comparable.

**Table 4:** Clinician-oriented summary of major interventions for hip-fracture prevention.

Intervention	Overall Strength of Evidence *	Main Benefits	Main Risks/Limitations	Best Target Population	Seminal Reference
Non-pharmacologic: long-term balance-challenging, resistance-based exercise	High	Reduces falls and injurious falls; probably contributes to fracture prevention; modestly improves femoral-neck and total-hip BMD	Hip fracture is rarely an isolated primary endpoint; effects depend on program content, dose, supervision, progression, and adherence	Community-dwelling older adults at fall risk; also appropriate after a sentinel fall when progression and support can be maintained	Long-term exercise meta-analysis [16]
Non-pharmacologic: targeted multifactorial/home-hazard intervention	Moderate	Can reduce fall rate when individualized assessment is linked to implementation; addresses environmental and clinical risk factors	Less consistent than exercise alone; depends heavily on patient selection, uptake, and intervention intensity	Older adults with prior falls, frailty, gait disorder, cognitive impairment, or clear home hazards	Tinetti multifactorial trial [28]
Non-pharmacologic: medication review/deprescribing as adjunct	Limited	Reduces exposure to fall-promoting medications; may strengthen broader fall-prevention programs	Limited benefit as a stand-alone strategy	Older adults with polypharmacy, sedatives, psychotropics, or drugs contributing to orthostatic symptoms	Deprescribing meta-analysis [40]
Hip protectors	Moderate in long-term care; limited in community	Probably reduce hip fractures when actually worn; useful adjunct in institutional settings	Adherence, discomfort, toileting/workflow barriers; no reliable community benefit	Frail ambulatory residents of long-term care/nursing homes at high fall risk	Cochrane review [43]
Calcium + vitamin D	Moderate (setting-specific)	Clearest hip-fracture benefit in frail institutionalized older adults with low intake or deficiency; attenuates femoral-neck bone loss	Community benefit is small or mixed and adherence-dependent; nephrolithiasis in some settings	Frail institutionalized older adults; patients with deficiency or low intake; adjunct to osteoporosis therapy	Decalyos I [67]
Vitamin D alone	Limited for fracture prevention	Corrects deficiency	No meaningful hip-fracture prevention in vitamin D-replete community-dwelling adults; high-dose bolus regimens may increase falls and fractures	Patients with documented vitamin D deficiency; not as stand-alone fracture prophylaxis	VITAL ancillary fracture trial [59]
Calcium alone	Limited	Small early gain in hip BMD	No convincing hip-fracture reduction; skeletal effect plateaus early	Patients with inadequate dietary calcium intake only	Systematic review [65]
Pharmacologic: bisphosphonates	High	Robust hip-fracture reduction in osteoporosis/high-risk patients; strong primary and secondary prevention evidence	Oral adherence and tolerability issues; rare atypical femoral fracture and osteonecrosis of the jaw with long-term use; duration reassessment is required	Patients with osteoporosis, prior fragility fracture, low femoral-neck BMD, or high fracture probability	HORIZON-PFT [80]
Pharmacologic: denosumab	High	Direct placebo-controlled hip-fracture reduction; progressive hip and femoral-neck BMD gains	Strict 6-month dosing is required; rebound bone loss and vertebral fractures can occur if delayed or stopped without sequential therapy	High-risk patients with poor oral adherence or bisphosphonate unsuitability	FREEDOM [83]
Pharmacologic: teriparatide	Moderate	Anabolic gains in femoral-neck and hip BMD; strong vertebral efficacy; useful in very-high-risk patients	Hip-fracture evidence is mainly indirect; requires subsequent antiresorptive consolidation	Very-high-risk osteoporosis when anabolic therapy is appropriate	Hip-fracture meta-analysis [91]
Pharmacologic: abaloparatide	Moderate	Rapid total-hip and femoral-neck BMD gains; strong vertebral and non-vertebral fracture reduction	Direct hip-fracture proof remains limited because hip events were rare in pivotal trials; requires antiresorptive consolidation	Very-high-risk postmenopausal osteoporosis when anabolic-first therapy is considered	ACTIVE trial [94]
Pharmacologic: romosozumab-based sequential therapy	High	Rapid hip BMD improvement; clearest direct hip-fracture RCT evidence among anabolic/dual-acting agents	Cardiovascular risk must be considered; should be followed by potent antiresorptive therapy	Very-high-risk or imminent-risk patients needing rapid hip protection	ARCH [103]
Pharmacologic: menopausal hormone therapy (MHT)	Moderate	Preserves hip BMD and reduces hip fractures during active treatment	Venous thromboembolism, stroke, breast cancer, and other systemic risks depending on regimen and patient profile; benefit wanes after cessation	Younger symptomatic postmenopausal women with a favorable overall risk profile	WHI estrogen plus progestin trial [171]
Pharmacologic: selective estrogen receptor modulators (SERMs)	Limited for hip-fracture prevention	Vertebral-fracture reduction; breast-risk reduction in selected women	Hip-fracture protection is not convincingly established; venous thromboembolism and hot flashes	Younger postmenopausal women with vertebral-predominant risk and/or a need for breast-risk reduction	MORE trial [192]
Secondary prevention: fracture liaison service (FLS)/coordinated post-fracture pathway	Moderate	Improves case findings, bone-health assessment, treatment initiation, and adherence; associated with lower secondary fragility fractures over time	Service models are heterogeneous; success depends on explicit accountability and follow-up	All adults after a fragility hip fracture	FLS meta-analysis [11]
Secondary prevention: default in-hospital initiation/order sets after hip fracture	Moderate	Improves real treatment delivery and persistence; may help prevent second fragility fractures	Requires inpatient workflow, named ownership before discharge, and reliable handover to outpatient follow-up	Hospitalized hip-fracture patients, especially very old adults at high risk of missed follow-up	Initial-hospitalization zoledronic acid study [216]

* Overall strength of evidence is a qualitative narrative judgment for this review and should not be interpreted as a formal GRADE assessment. Abbreviations: BMD = bone mineral density; FLS = fracture liaison service; MHT = menopausal hormone therapy; SERM = selective estrogen receptor modulator. Reference numbers in the “Seminal reference” column correspond to the current manuscript numbering.

## Data Availability

No new data were created or analyzed in this study. Data sharing is not applicable to this article.
